# Age-related declines in α-Klotho drive progenitor cell mitochondrial dysfunction and impaired muscle regeneration

**DOI:** 10.1038/s41467-018-07253-3

**Published:** 2018-11-19

**Authors:** A. Sahu, H. Mamiya, S. N. Shinde, A. Cheikhi, L. L. Winter, N. V. Vo, D. Stolz, V. Roginskaya, W. Y. Tang, C. St. Croix, L. H. Sanders, M. Franti, B. Van Houten, T. A. Rando, A. Barchowsky, F. Ambrosio

**Affiliations:** 1 Department of Physical Medicine and Rehabilitation, University of Pittsburgh, Pittsburgh, 15213 PA USA; 2 Department of Environmental and Occupational Health, University of Pittsburgh, Pittsburgh, 15261 PA USA; 3 Department of Bioengineering, University of Pittsburgh, Pittsburgh, 15260 PA USA; 4 Division of Geriatric Medicine, Department of Medicine, University of Pittsburgh, Pittsburgh, 15213 PA USA; 5 Department of Orthopaedic Surgery, University of Pittsburgh, Pittsburgh, 15213 PA USA; 6 Department of Pathology, University of Pittsburgh, Pittsburgh, 15261 PA USA; 7 Department of Cell Biology, University of Pittsburgh, Pittsburgh, 15261 PA USA; 8 Department of Pharmacology & Chemical Biology, University of Pittsburgh Cancer Institute, University of Pittsburgh, Pittsburgh, 15232 PA USA; 9 Department of Environmental Health and Engineering, Johns Hopkins Bloomberg School of Public Health, Baltimore, 21218-2608 MD USA; 10 Department of Neurology, Duke University School of Medicine, Durham, 27704 NC USA; 11 Research Beyond Borders: Boehringer-Ingelheim Pharmaceuticals, Ridgefield, 06877 CT USA; 12 Department of Neurology and Neurological Sciences, Stanford University School of Medicine, Stanford, CA 94305 USA; 13 The Paul F. Glenn Center for the Biology of Aging, Stanford University School of Medicine, Stanford, CA 94305 USA; 14 Center for Tissue Regeneration, Restoration and Repair, Veterans Affairs Hospital, Palo Alto, CA 94036 USA; 15 McGowan Institute for Regenerative Medicine, University of Pittsburgh, Pittsburgh, 15219 PA USA

## Abstract

While young muscle is capable of restoring the original architecture of damaged myofibers, aged muscle displays a markedly reduced regeneration. We show that expression of the “anti-aging” protein, α-Klotho, is up-regulated within young injured muscle as a result of transient *Klotho* promoter demethylation. However, epigenetic control of the *Klotho* promoter is lost with aging. Genetic inhibition of α-Klotho in vivo disrupted muscle progenitor cell (MPC) lineage progression and impaired myofiber regeneration, revealing a critical role for α-Klotho in the regenerative cascade. Genetic silencing of *Klotho* in young MPCs drove mitochondrial DNA (mtDNA) damage and decreased cellular bioenergetics. Conversely, supplementation with α-Klotho restored mtDNA integrity and bioenergetics of aged MPCs to youthful levels in vitro and enhanced functional regeneration of aged muscle in vivo in a temporally-dependent manner. These studies identify a role for α-Klotho in the regulation of MPC mitochondrial function and implicate α-Klotho declines as a driver of impaired muscle regeneration with age.

## Abstract (web summary)

While young muscle faithfully regenerates damaged myofibers, aged muscle is impaired. Here the authors show the “anti-aging” protein α-Klotho is upregulated in young muscle after damage via promoter demethylation and this regulation is lost in aging, resulting in mitochondrial damage and an impaired healing response.

## Introduction

Aging is associated with impaired skeletal muscle regenerative capacity after an acute injury, resulting in declines in force-producing capacity. The impaired regenerative response of aged muscle is characterized by a shift from functional myofiber repair following injury to fibrotic deposition^1^. This increased fibrosis has been attributed to muscle stem (satellite) cell (MuSCs) dysfunction^1^.

In response to muscle injury, MuSCs become activated from a quiescent state to repair damaged myofibers^2,3^. While MuSC activation in young muscle restores the original architecture of the damaged myofibers, aging is associated with MuSC dysfunction, as evidenced by increased apoptosis^4^, decreased proliferation^5^, impairment of autophagy^6^, and a decreased resistance to stress^7^. Aged MuSCs also display a manifold increase in expression of aging-associated senescence markers, including p16^Ink4a^ and p21^Cip18^. Though aged MuSCs clearly display cell-autonomous deficits that contribute to an impaired regenerative response^8–11^, it was recently suggested that extrinsic changes in the muscle microenvironment may provide the initial geroconversion trigger in MuSCs^12^. Indeed, several studies have demonstrated that rejuvenation of the systemic muscle microenvironment largely restores the healing capacity of aged skeletal muscle^1,5,13,14^, leading to interest in the identification of circulating “anti-geronic” proteins and an improved mechanistic understanding by which such proteins may transpose a youthful regenerative phenotype onto aged skeletal muscle.

To this end, genetic studies have identified a powerful aging suppressor gene, *Klotho*, which encodes a membrane-bound and circulating hormonal protein in mice and humans^15,16^. Klotho deficiency results in the onset of numerous aging phenotypes, including decreased activity levels, gait disturbances, cognitive impairment, sarcopenia, as well as an impaired wound repair process^15,17–22^. Three homologs of Klotho, α, β, and γ, have been identified (reviewed in ref. ^23^). In tissues such as the skin, small intestine, and kidney, declines in α-Klotho have been shown to promote cellular senescence^24^ and stem cell dysfunction^17^.

In this study, we tested the hypothesis that age-related declines in α-Klotho drive dysfunctional muscle progenitor cell (MPC) mitochondrial bioenergetics, ultimately resulting in an impaired tissue regeneration. Our findings demonstrate that young skeletal muscle displays a robust increase in local α-Klotho expression following an acute muscle injury with transient demethylation of the *Klotho* promoter. However, aged muscle displays no change in *Klotho* promoter methylation and no increase in α-Klotho expression following injury. Levels of α-Klotho in MPCs derived from aged mice are decreased relative to those of young animals, and genetic knockdown of α-Klotho in young MPCs confers an aged phenotype with pathogenic mitochondrial ultrastructure, decreased mitochondrial bioenergetics, mitochondrial DNA damage, and increased senescence. Further supporting a role for α-Klotho in skeletal muscle vitality, mice heterozygously deficient for Klotho (*Kl*^*+/−*^) have impaired MPC bioenergetics that is consistent with a defective regenerative response following injury, but the regenerative defect of *Kl*^*+/−*^ mice is rescued at the cellular and organismal level when mitochondrial ultrastructure is restored through treatment with the mitochondria-targeted peptide, SS-31^25^. Finally, we demonstrate that systemic delivery of exogenous α-Klotho rejuvenates MPC bioenergetics and enhances functional myofiber regeneration in aged animals in a temporally dependent manner. Together, these findings reveal a role for α-Klotho in the regulation of MPC mitochondrial function and skeletal muscle regenerative capacity.

## Results

### Aged muscle displays a blunted α-Klotho response to injury

**Fig. 1:**
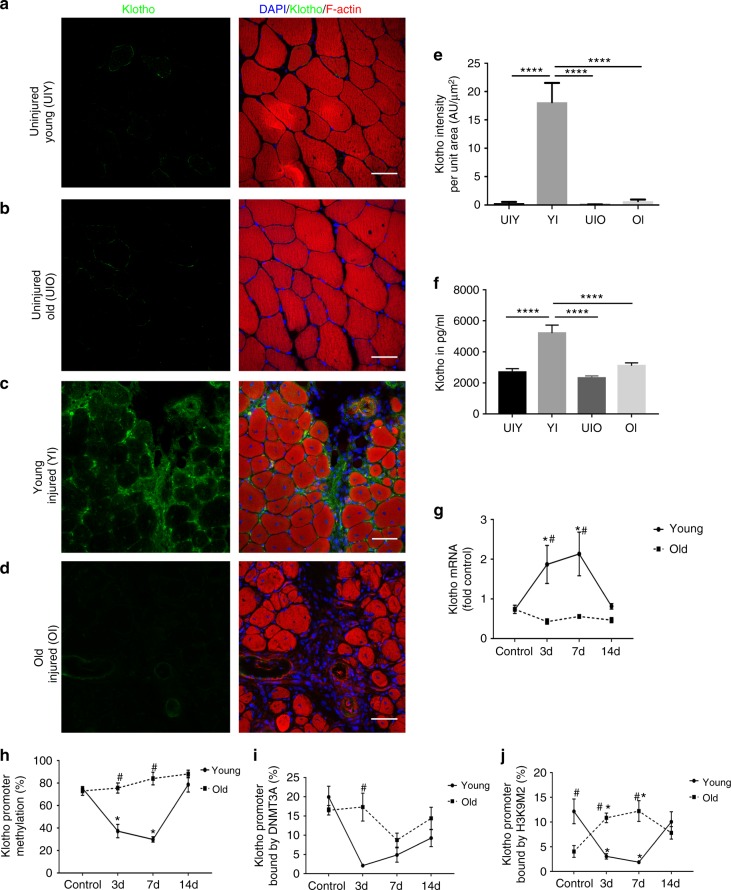
α-Klotho is increased in young muscle after injury, but the response is lost  with increased age. **a**−**d** Immunofluorescent imaging of α-Klotho and F-actin in skeletal muscle from uninjured young (UIY; 4–6 months) and old mice (UIO; 22–24 months) as well as 14 days post injury (dpi) in young (YI) and old (OI) mice. Scale: 50 µm. **e** Quantification of α-Klotho across the four comparison groups, UIY, UIO, YI, and OI. Experimental cohorts were performed in duplicate, *n* = 3/group/cohort. ****p < 0.0001. **f** ELISA analysis of serum obtained from UIY, UIO, YI, and OI mice (*n* = 8–11/group; ****p < 0.0001). **g** RT-qPCR analysis of α-Klotho in young and old muscles at 0 (control), 3, 7, and 14 dpi (*n* = 4/age/timepoint). **h** MSPCR analysis of young and old muscle at 0 (control), 3, 7, and 14 dpi (*n* = 4/age/timepoint). **i** ChIP analysis of DNMT3a in young and old muscle at 0 (control), 3, 7, and 14 dpi (*n* = 3-4/age/timepoint). **j** ChIP analysis of H3K9me2 in the *Klotho* promoter in young and old muscle at 0 (control), 3, 7, and 14 dpi (*n* = 4/age/timepoint) (*p < 0.05 compared to young, sex-matched uninjured muscles; #*p* < 0.05 indicates a significant difference between young and aged groups at the respective timepoint). **e**−**g** One-way ANOVA with Tukey’s post-hoc test. **h**−**j** Two-way ANOVA with Tukey’s post-hoc test. Data represented as mean ± SEM

To determine whether α-Klotho is upregulated locally in response to an acute muscle injury, we performed immunofluorescence analysis of α-Klotho in the skeletal muscle of young (4–6 months) and aged (22–24 months) male mice under conditions of homeostasis and following a cardiotoxin-induced injury. α-Klotho was virtually undetectable in healthy, uninjured muscle, regardless of age (Fig. 1a–e). In contrast, strong expression of α-Klotho was observed at the regenerating site of young muscle 14 days post injury (dpi) (Fig. 1c, e; confirmation of antibody specificity is presented in Supplementary Fig. 1). Aged muscle, however, displayed no appreciable increase in α-Klotho expression following an acute injury (Fig. 1d, e). Serum α-Klotho levels followed a similar expression pattern according to age and injury status (Fig. 1f). RT-qPCR findings revealed that *Klotho* transcript expression increases significantly at 3 and 7 dpi injury in the skeletal muscle of young mice (Fig. 1g). Despite the fact that α-Klotho protein is still detected in young muscle at 14 dpi (Fig. 1c, e), gene expression approached baseline levels at this later time point. On the other hand, aged counterparts display unaltered gene expression across all the time points tested (Fig. 1g). The α-Klotho response to injury was not unique to a cardiotoxin injury, as we found that young mice exposed to a severe contusion injury displayed a robust α-Klotho response at the protein level 14 days after injury (Supplementary Fig. 2). Young female mice displayed a similar, yet blunted, increase in *Klotho* expression in response to injury, but, like males, the response is lost with aging (Supplementary Fig. 3).

Epigenetic silencing of *Klotho* contributes to the impaired regenerative potential of dystrophic skeletal muscle, and a differentially methylated region (DMR) of 110 nucleotides within the *Klotho* promoter region was identified in the muscles of aged *mdx* mice^26^. Therefore, we measured methylation levels of the DMR after injury in young and aged muscle. An acute injury to young muscle triggered demethylation of the DMR in the *Klotho* promoter 3 and 7 dpi (Fig. 1h). Injury-induced demethylation was, however, absent in the *Klotho* promoter within aged muscle (Fig. 1h).

To examine whether modifying enzymes for DNA methylation contribute to the observed methylation changes, we used a chromatin immunoprecipitation (ChIP) assay to measure the enrichment of DNA methyltransferases 3a (DNMT3a) in the *Klotho* promoter region^26^. There was a decrease of DNMT3a binding to the *Klotho* promoter in the injured young muscle that, at the timepoints evaluated, reached a nadir 3 days after injury and was increased by day 14 (Fig. 1i). In contrast, the injury-induced decrease in DNMT3a binding was delayed and blunted in injured aged muscle (Fig. 1i). Consistent with the finding that dimethylated histone H3 lysine 9 (H3K9me2) bound to the *Klotho* promoter in dystrophic skeletal muscle represses *Klotho*^26^, H3K9me2 binding to the *Klotho* promoter region was decreased after injury only in young muscle (Fig. 1j). Taken together, these findings suggest that acute injury drives the reactivation of *Klotho* by reducing DNA methylation and H3K9 dimethylation in the promoter of young muscle, but that *Klotho* remains epigenetically repressed after injury in aged muscle.

### Genetic inhibition of α-Klotho impairs muscle regeneration

**Fig. 2:**
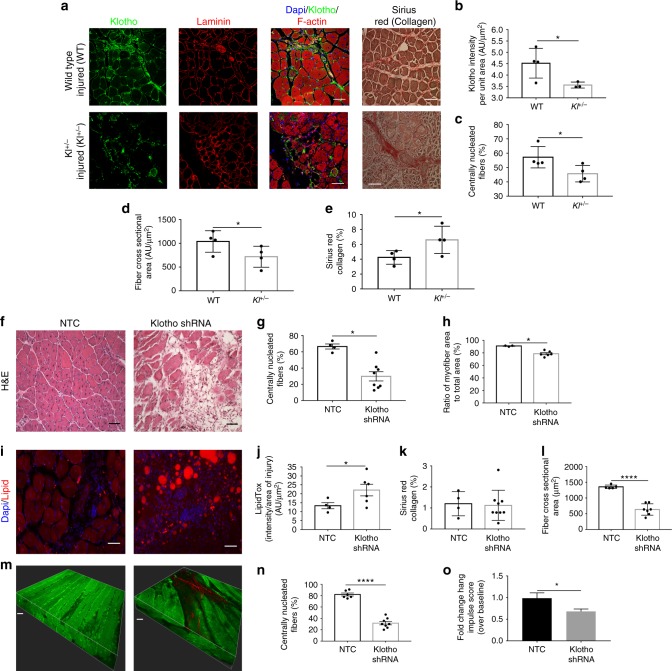
Genetic and muscle-specific loss of α-Klotho impairs skeletal muscle regeneration. **a** Immunofluorescence of α-Klotho, laminin, F-actin, and Sirius red stain in wild-type and *Kl*^*+/−*^ mice 14 dpi. Scale: 50 µm. **b** Quantitation of α-Klotho in wild-type versus *Kl*^*+/−*^ mice 14 dpi (*n* = 3–4/group; *p < 0.05, one-tailed Student’s *t* test). **c**−**e** Quantitation of the % of centrally nucleated fibers (*n* = 4/group; *p < 0.05, Mann−Whitney U test), fiber cross-sectional area (*n* = 4/group; *p < 0.05, one-tailed Student’s *t* test) and collagen (Sirius red) deposition (*n* = 4/group; *p < 0.05, Welchʼs *t* test). **f** Representative hematoxylin and eosin stain of non-targeting control (NTC) and shRNA to α-Klotho (0.2–3.82×10^6^ TU/TA) Scale: 50 µm. **g**, **h** Quantification of the % centrally nucleated fibers and ratio of myofiber area to total area, respectively, in NTC and Klotho shRNA-treated mice at 14 dpi (*n* = 3–8/group; *p < 0.05, Mann−Whitney U test). **i** Representative immunofluorescence imaging of lipid in NTC and α-Klotho shRNA-treated muscle at 14 dpi. Scale: 50 µm. **j** Quantification of lipid in NTC and Klotho shRNA-treated muscle 14 dpi (*n* = 4–6/group; *p < 0.05, one-tailed Student’s *t* test). **k** Quantification of collagen deposition (Sirius red) in NTC and Klotho shRNA groups (*n* = 4–9/group; *p* > 0.05, Student’s *t* test). **l** Quantification of fiber cross-sectional area of regenerating muscle fibers in NTC and α-Klotho shRNA-treated muscle (*n* = 5–7/group; ****p < 0.0001, one-tailed Student’s *t* test). **m** Representative second harmonic generation (SHG) images of tibialis anterior (TA) muscles injected with NTC or α-Klotho shRNA. Scale: 30 µm. **n** SHG quantification of the regeneration index in NTC and Klotho shRNA-treated mice at 14 dpi (*n* = 6–8/group; ****p < 0.0001, one-tailed Student’s *t* test). **o** Hang impulse (calculated as hanging time × mouse weight) at 14 dpi represented as a fold  change from baseline score pre-injury (*n* = 6/group; *p < 0.05, one-tailed Student’s *t* test). Data represented as mean ± SEM

To directly implicate a functional role for α-Klotho in skeletal muscle regeneration, we next evaluated the regenerative response to acute muscle injury in adult mice that are heterozygously deficient for Klotho (*Kl*^*+/−*^ mice). As expected, *Kl*^*+/−*^ mice displayed a significantly decreased local α-Klotho expression at the site of injury (Fig. 2a, b). *Kl*^*+/−*^ mice also displayed a decreased regenerative index, smaller myofiber cross-sectional area, and increased fibrosis when compared to age- and sex-matched wild-type counterparts (Fig. 2a−e). These findings are consistent with a recent study of muscle regeneration in α-Klotho hypomorphs^27^. Together, the data demonstrate that α-Klotho is necessary for effective skeletal muscle regeneration after injury.

Given our findings of increased *Klotho* expression within the injured muscles of young mice (Fig. 1c,g,e), we next evaluated the contribution of local α-Klotho in functional muscle regeneration. The tibialis anterior (TA) muscles of young mice were injected with GFP-tagged SMARTpool® lentiviral particles carrying shRNA to α-Klotho, whereas control counterparts were injected with an equal volume of the non-targeting control (NTC) lentivirus. After 4 weeks, TAs were injured via a local cardiotoxin injection, and regeneration was evaluated 2 weeks after injury. As expected, muscles treated with shRNA to α-Klotho displayed a significant decrease in α-Klotho expression at the site of injury (Supplementary Fig. 1B, C). Circulating α-Klotho was also significantly decreased (Supplementary Fig. 1D). This suggests that muscle-derived α-Klotho may contribute to the increased circulating levels observed in young mice after injury (Fig. 1f).

Histological analysis revealed that knockdown of α-Klotho expression resulted in a decreased number of regenerating fibers, a decrease in the percentage of myofiber area/total area, and increased adiposity (Fig. 2f−j). There was, however, no difference in the fibrosis across groups (Fig. 2k). The cross-sectional area of regenerating (centrally nucleated) fibers in muscles treated with shRNA to α-Klotho was also significantly smaller than NTC controls (Fig. 2l), further confirming a defective regenerative response. Unexpectedly, we also observed the presence of a number of large, nonregenerating myofibers at the injury site of muscles treated with shRNA to α-Klotho (Fig. 2f). To evaluate the structural integrity of these myofibers, we performed second harmonic generation (SHG) imaging, which allows for three-dimensional visualization of myofiber structure and organization. Consistent with histological findings, SHG analysis revealed that muscles treated with shRNA to α-Klotho contain a decreased number of centrally nucleated fibers (Fig. 2m, n). This decreased evidence of active regeneration was concomitant with pathologic myofiber architecture and integrity (Fig. 2m and Supplementary Movies 1 and 2). Most importantly, the impaired regenerative response and disrupted myofiber structure was concomitant with a decreased functional recovery after injury (Fig. 2o).

### α-Klotho is expressed by MuSCs and their progeny

**Fig. 3:**
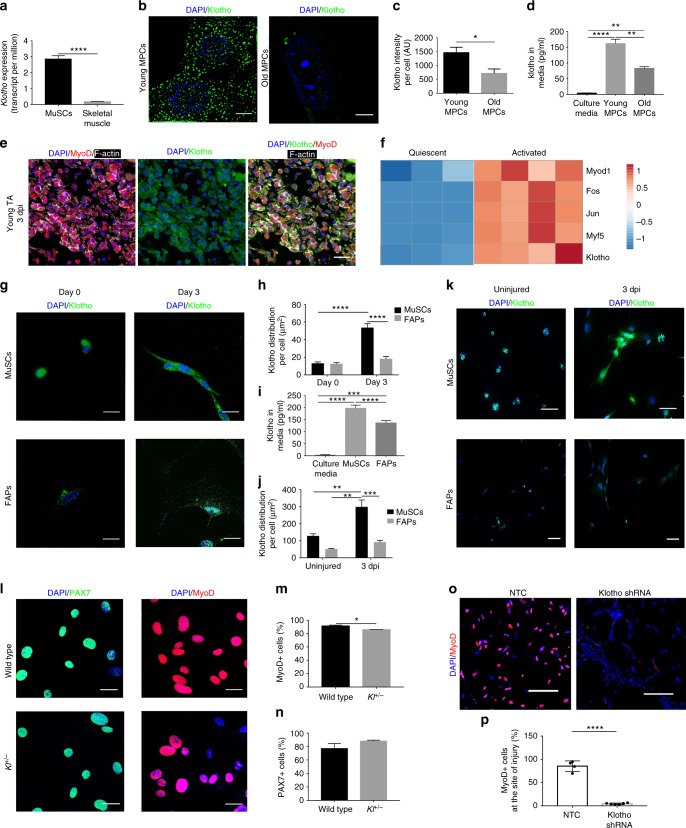
α-Klotho expression in quiescent and activated MuSCs. **a** *Klotho* expression in isolated MuSCs versus whole muscle lysates as per RNAseq analysis in transcripts per million (TPM)^27^ (*n* = 4/group; ****p < 0.0001, one-tailed Student’s *t* test). **b** Representative structured illuminescent microscopy of α-Klotho in young and old MPCs. Scale: 5 µm. **c** Quantification of α-Klotho in young and old MPCs (*p < 0.05, one-tailed Student’s *t* test). **d** ELISA analysis of α-Klotho in culture media alone, as well as conditioned media from young and old MPCs (*n* = 3/group; **p < 0.01, ****p < 0.0001, one-way ANOVA with Tukey’s post-hoc test). **e** Immunofluorescent colocalization of MyoD, F-actin, and α-Klotho 3 dpi. Scale: 50 µm. **f** Heat-map representation of α-Klotho as well as markers of MuSC activation (*MyoD1, Fos, Jun, Myf5*) in quiescent and activated cells from RNASeq analysis of publicly archived data from a recent report^27^. **g** Immunofluorescence staining of α-Klotho and DAPI in sorted MuSCs and fibroadipogenic progenitors (FAPs) fixed immediately after isolation (Day 0) or after activation in culture (Day 3). Scale: 12.5 µm. **h** Quantification of α-Klotho expression in MuSCs and FAPs at Day 0 and Day 3 of culture (****p < 0.0001, two-way ANOVA with Tukey’s post-hoc test). **i** Quantification of α-Klotho in the conditioned media of MuSCs and FAPs sorted from uninjured muscle. Conditioned media was obtained after 3 days in culture (*n* = 3/group; ****p < 0.0001, one-way ANOVA with Tukey’s post-hoc test). **j** Quantification of α-Klotho in MuSCs and FAPs isolated from uninjured muscle and muscle 3 dpi (**p < 0.01, ***p < 0.001, two-way ANOVA with Tukey’s post-hoc test). **k** Immunofluorescence imaging of α-Klotho and DAPI in MuSCs and FAPs sorted from uninjured muscle and muscle 3 dpi. Scale: 50 µm. **l** Immunofluorescent imaging of Pax7 and MyoD in MPCs isolated from wild-type and *Kl*^*+/−*^ mice. Scale: 50 µm. **m** Quantification of the % of MyoD+ cells in MPCs from wild-type and *Kl*^*+/−*^ mice (*p < 0.05, one-tailed Student’s *t* test). **n** Quantification of the % of Pax7+ cells in MPCs from wild-type and *Kl*^*+/−*^ mice (One-tailed Student’s *t* test). **o** Immunofluorescent imaging of MyoD and DAPI in the injured muscles of nontargeting control (NTC) and shRNA to α-Klotho 14 dpi. Scale: 25 µm. **p** Quantification of the percentage of MyoD+ nuclei within the injured muscles of nontargeting control (NTC) and shRNA to α-Klotho 14 dpi (*n* = 4–6/group; ****p < 0.0001, one-tailed Student’s *t* test). Data represented as mean ± SEM

The fact that α-Klotho expression in young muscle is elevated at 3 days after injury (Fig. 1g)—a time point that corresponds with MuSC activation—led us to next investigate whether MuSCs express α-Klotho and whether α-Klotho is necessary for the MuSC response to injury. We accessed RNAseq data from a recent study^28^, which is stored on the Gene Expression Omnibus (GEO) publicly accessible database. Analysis of archived data revealed a ten-fold increase in *Klotho* expression of freshly sorted MuSCs as compared to whole muscle lysates (Fig. 3a). Structured illumination microscopy (SIM) confirmed robust α-Klotho in MPCs isolated from young mice (Fig. 3b, c). MPCs were cultured for no more than three passages prior to analysis and were confirmed to be >90% MyoD^+^. MPCs isolated from aged muscle, however, displayed a markedly decreased α-Klotho protein expression (Fig. 3b, c). We also evaluated α-Klotho expression in MuSCs isolated by fluorescence-activated cell sorting^29^. As observed in MPCs, we found that young MuSCs displayed a robust α-Klotho expression, but that α-Klotho expression was decreased in aged MuSCs (Supplementary Fig. 4).

Tissues previously shown to express high levels of α-Klotho, such as the kidney, contain a membrane-bound form of α-Klotho (~120 kDa), which functions as an obligate co-receptor for fibroblast growth factor-23 (FGF23)^30^. However, upon proteolytic cleavage of the extracellular domain, α-Klotho is released from the cells, where it functions as a humoral factor^15^. To determine whether MPCs secrete α-Klotho, we performed ELISA of the conditioned media from MPCs isolated from young and old muscle. Conditioned media derived from young MPCs contained significantly more α-Klotho than the conditioned media from aged MPCs (Fig. 3d), suggesting that MuSCs secrete α-Klotho but that secretion declines with age.

Immunohistochemical analysis of young muscle three days after injury revealed that 96.7% of MyoD^+^ cells express α-Klotho (Fig. 3e). MyoD is a marker of activated MuSCs and is required for myogenic lineage progression^31^. We accessed and analyzed MuSCs RNAseq data housed on the GEO repository^28^ and found that activated MuSCs display significantly increased *Klotho* expression when compared to quiescent MuSC counterparts that were isolated from 1% PFA-perfused mice (Fig. 3f). These findings suggest that α-Klotho expression is increased in the transition from MuSC quiescence to activation. To investigate this more directly, we compared α-Klotho expression in freshly sorted (quiescent) MuSCs and MuSCs that were maintained in culture for 3 days, thereby promoting activation in vitro^32^. Activated MuSCs displayed a 4.5-fold increase in α-Klotho, when compared to quiescent counterparts (Fig. 3g, h). We also isolated MuSCs from uninjured young muscle and young muscle 3 dpi, yielding a population of quiescent and activated MuSCs, respectively (Supplementary Fig. 4C). As observed when MuSCs were activated in vitro, there was a significant increase in α-Klotho expression in MuSCs that were activated in vivo (Fig. 3j, k). As a comparison, fibroadipogenic progenitor cells (FAPs), which also play a critical role in the skeletal muscle regenerative cascade^33^, displayed no change in α-Klotho over 3 days of activation in culture, nor was α-Klotho expression increased when FAPS were isolated from acutely injured muscle (Fig. 3g–k, Supplementary Fig. 4D). The conditioned media from activated FAPs also contained significantly less α-Klotho when compared to the conditioned media from activated MuSCs (Fig. 3i). Therefore, at least some of the α-Klotho protein detected in muscle after injury could come from MPCs themselves, although other neighboring cell populations may also express and secrete α-Klotho in response to an acute injury. Indeed, it was recently demonstrated that α-Klotho derived from macrophages promotes muscle regeneration^34^.

We next asked whether α-Klotho is necessary for normal MuSC lineage progression. MPCs isolated from the skeletal muscle of *Kl*^*+/−*^ mice displayed a small, but significant, decrease in the percentage of MyoD^+^ cells when compared to age-matched wild-type counterparts. There was, however, no difference in Pax7 expression across groups (Fig. 3l–n). These findings are consistent with a previous report demonstrating that α-Klotho hypomorphs display a decreased number of MyoD^+^ cells as compared to control counterparts^27^. In vivo, lentiviral shRNA inhibition of α-Klotho resulted in a decreased MyoD expression at the site of injury (Fig. 3o, p). Taken together, these data suggest that a loss of α-Klotho disrupts MuSC lineage progression.

### Loss of α-Klotho in MPCs drives mitochondrial dysfunction

Previous studies have implicated α-Klotho as being involved in multiple processes associated with inhibition of senescence^24^. Senescence is actively repressed in young MuSCs, but this capacity declines with aging, resulting in a failure of MuSCs to activate and proliferate in response to injury^35^. Therefore, we investigated whether the decreased MuSC activation in α-Klotho-deficient muscles may be attributed to cellular senescence. To confirm an inhibitory role for α-Klotho in myogenic cell senescence, we used small interference RNA (siRNA) inhibition, which resulted in a ~3-fold decrease in α-Klotho (Supplementary Fig. 1G, H). As expected, knockdown of α-Klotho induced a senescent phenotype in young MPCs, as evidenced by the percentage of senescence associated (SA)-βgal-positive cells, increased cytosolic HMGB1 levels (an indicator of cellular stress), and decreased cellular proliferation (Supplementary Fig. 5). These findings mimicked the phenotype of MPCs isolated from aged muscle (Supplementary Fig. 5).

Given that senescent cells are known to have higher levels of DNA damage^36^, we used LXRepair multiplex technology^37,38^ to evaluate the DNA base excision repair (BER) enzyme activities of OGG1 and APE1, which work on two common oxidative DNA lesions, 8-oxodG and abasic sites, respectively. When compared to scrambled siRNA-treated young MPCs, there was no significant decline in BER in the nucleus of cells treated with siRNA to α-Klotho (Supplementary Fig. 5G, H). However, just 48 h of α-Klotho supplementation to siRNA-treated MPCs from young animals dramatically stimulated the activity of these BER enzymes (Supplementary Fig. 5G, H). These data suggest that α-Klotho’s role in MPC senescence may be attributed to induction of key enzymes in the BER pathway, which is rapid and can occur even within a couple of hours^39^.

**Fig. 4:**
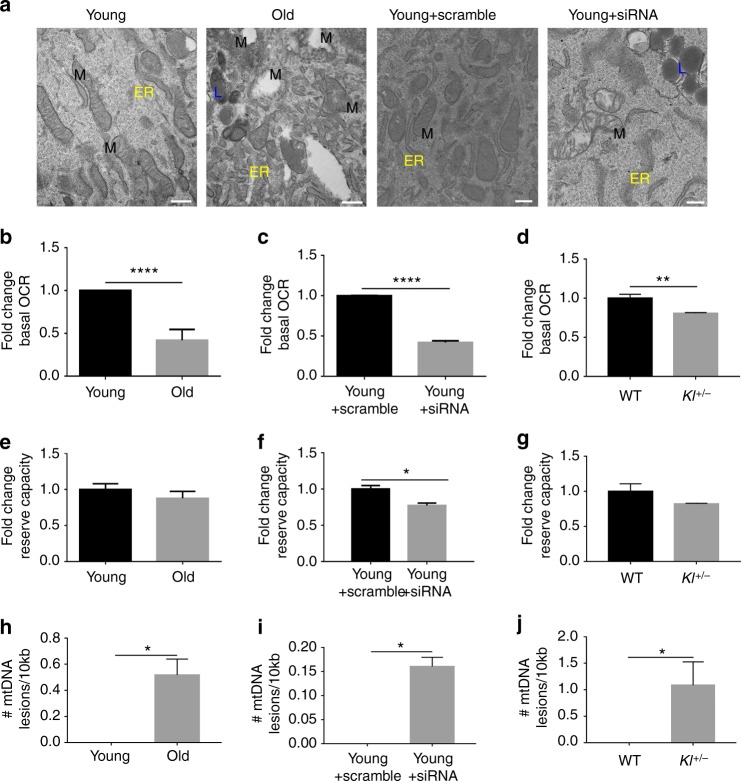
Loss of α-Klotho drives mitochondrial dysfunction and disrupts mitochondrial DNA integrity. **a** TEM images of young, old, young + scramble and young + siRNA MPCs showing mitochondria (M), lipid droplet accumulation (L), as well as endoplasmic reticuli (ER). Scale: 400 nm. **b**, **c** Seahorse analysis of young, old, young + scramble and young + siRNA MPCs quantifying the basal oxygen consumption rate (OCR). **d** Seahorse analysis of basal OCR of MPCs isolated from wild-type and *Kl*^*+/−*^ mice. **e**, **f** Seahorse analysis of reserve capacity (calculated as the difference between basal and maximum OCR) of young, old, young + scramble and young + siRNA MPCs. **g** Seahorse analysis of reserve capacity of MPCs isolated from wild-type and *Kl*^*+/−*^ mice. **h**, **i** RT-qPCR based analysis of mtDNA damage in young, old, young + scramble and young + siRNA MPCs. **j** RT-qPCR analysis of mtDNA damage in MPCs isolated from wild-type and *Kl*^*+/−*^ mice (*p < 0.05, **p < 0.01, ****p < 0.0001, one-tailed Student’s *t* test). Data represented as mean ± SEM

While α-Klotho’s role in cellular senescence has been demonstrated in multiple systems, the mechanisms underlying this role are incompletely understood. Cellular senescence is associated with altered mitochondrial morphology and dysregulated bioenergetics that can lead to aging and aging-associated pathologies^40,41^. Thus, regulation of MPC senescence by α-Klotho raised the intriguing possibility that α-Klotho may regulate MPC mitochondrial structure and function. Using an antibody against the mitochondrial membrane protein, Tom20, we observed no difference in mitochondrial morphology, as determined by sphericity, the number of mitochondria per cell, or mitochondrial volume according to age or α-Klotho levels (Supplementary Fig. 6A, C–F). This was further supported qualitatively using STimulation Emission Depletion (STED) microscopy to visualize the mitochondrial network (Supplementary Fig. 6B). These findings suggest that a loss of α-Klotho does not affect mitochondrial morphology or mass. However, detailed analysis by transmission electron microscopy (TEM) revealed a striking alteration of ultrastructural integrity of the mitochondrial cristae and endoplasmic reticulum in addition to lipid droplet accumulation when young MPCs were treated with siRNA to α-Klotho (Fig. 4a).

In light of the loss of mitochondrial ultrastructure resulting from decreased α-Klotho expression, we next evaluated whether loss of α-Klotho drives MPC mitochondrial dysfunction. Indeed, recent reports have shown a critical role for mitochondria in MuSC function^42,43^. The bioenergetic profiles of young and aged MPCs were studied using a Seahorse XF^e^96 Flux analyzer, which quantifies the oxygen consumption rate (OCR), a measure of oxidative phosphorylation. OCR was measured again after sequential injection of oligomycin, FCCP, 2-deoxyglucose (2-DG), and rotenone. These data demonstrate that, when normalized to total number of cells, MPCs from older animals display dramatically decreased levels of basal OCR as compared to young counterparts (Fig. 4b; Supplementary Fig. 7A, B). A similar age-associated deficit in the OCR of freshly isolated and cultured MuSCs was recently reported^44^. When young MPCs were treated with siRNA to α-Klotho, basal OCR was reduced to ~25% values of scramble-treated counterparts (Fig. 4c, Supplementary Fig. 7A, B). MPCs isolated from uninjured *Kl*^*+/−*^ mice display a similarly blunted bioenergetic profile (Fig. 4d). Though there was no appreciable decrease in reserve capacity in aged MPCs, young MPCs treated with siRNA to α-Klotho and MPCs isolated from *Kl*^*+/−*^ mice both showed a ~25% decrease in the reserve capacity (Fig. 4e–g; Supplementary Fig. 8A). Reserve capacity represents the spare bioenergetic capacity, is calculated as the difference between the basal and maximal OCR, and indicates the ability of a cell to respond to stress^45^. Taken together, these data support the hypothesis that age-related declines in α-Klotho drive impaired MPC mitochondrial bioenergetics.

One factor that could contribute to decreased mitochondrial function with decreased α-Klotho is oxidative damage, which could manifest itself as mtDNA damage^46^. Therefore, we next examined mtDNA integrity. Using a qPCR-based assay that we have successfully applied to different cell types including mouse tissue and human peripheral lymphocytes^47–49^, we evaluated mtDNA damage in MPCs isolated from young or aged mice. The method used is based on the principle that a wide variety of types of DNA damage have the propensity to block DNA polymerase progression^46^. Therefore, this assay detects numerous kinds of base DNA damage or DNA repair intermediates such as abasic sites, as well as single and double DNA strand breaks.

There was no difference in steady-state mtDNA copy number across groups (Supplementary Fig. 7C, D), consistent with immunocytochemical analysis showing no difference in mitochondrial mass (Supplementary Fig. 6C). However, we found that MPCs isolated from aged mice displayed higher levels of mtDNA damage as compared to young counterparts (Fig. 4h). Accordingly, young MPCs receiving siRNA to α-Klotho and MPCs isolated from *Kl*^*+/−*^ mice displayed increased numbers of mtDNA lesions when compared to scramble-treated and wild-type controls, respectively (Fig. 4i, j).

### Loss of α-Klotho impairs mitochondrial bioenergetics

**Fig. 5:**
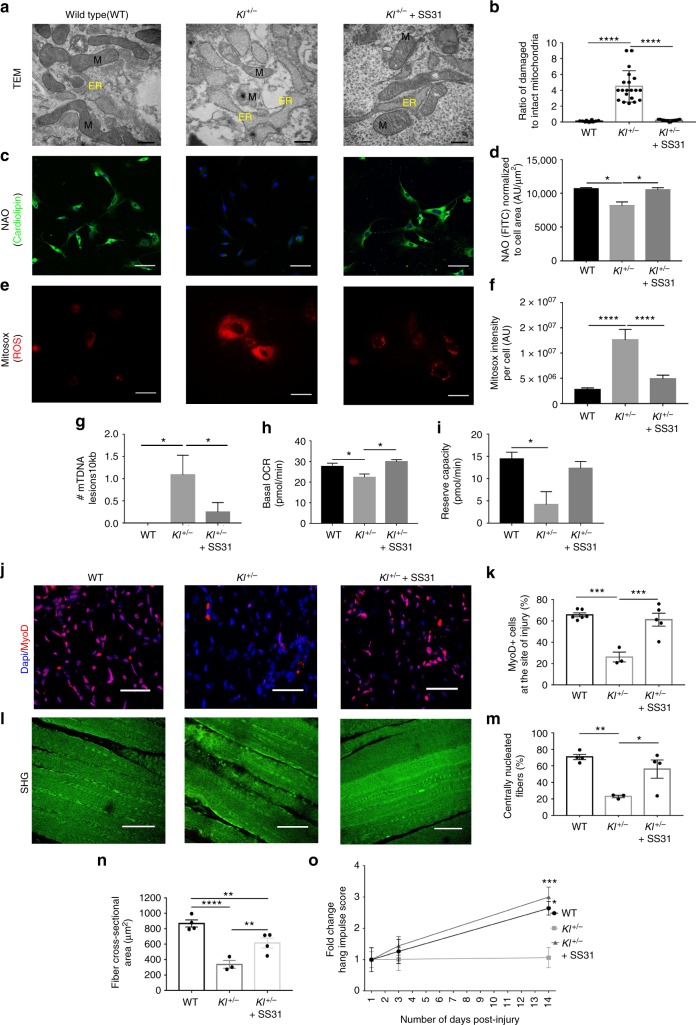
Mitochondrial structure and function are impaired in *Kl*^*+/−*^ mice, but are rescued with SS-31. **a**, **b** Representative TEM images and analysis of damaged mitochondria of wild-type (WT), *Kl*^*+/−*^ and *Kl*^*+/−*^+ SS-31 groups (10–20 images were analyzed to quantify >100 mitochondria/group; ****p < 0.0001, one-way ANOVA with Tukey’s post-hoc test). Scale: 500 nm. **c**, **d** Representative immunofluorescent images and quantification of cardiolipin content, by Nonyl Acridine Orange staining (NAO, green) in WT, *Kl*^*+/−*^ and *Kl*^*+/−*^+ SS-31 MPCs (*p < 0.05, one-way ANOVA with Tukey’s post-hoc test). Scale: 50 µm. **e**, **f** Representative immunofluorescent images from live imaging and quantification of ROS as determined by MitoSox staining (red) on live cells from WT, *Kl*^*+/−*^ and *Kl*^*+/−*^+ SS-31 groups (****p < 0.0001, one-way ANOVA with Tukey’s post-hoc test). Scale: 50 µm. **g** RT-qPCR-based analysis of mtDNA damage on WT, *Kl*^*+/−*^ and *Kl*^*+/−*^+ SS-31 MPCs (*n* = 3/group; *p < 0.05, one-way ANOVA with Tukey’s post-hoc test). **h**, **i** Seahorse analysis of basal OCR and reserve capacity of WT, *Kl*^*+/−*^ and *Kl*^*+/−*^+ SS-31 MPCs (*n* = 4–6/group; *p < 0.05, one-way ANOVA with Tukey’s post-hoc test). **j**, **k** Quantification of MyoD^+^ cells at the site of injury of TA muscles from WT, *Kl*^*+/−*^ and *Kl*^*+/−*^+ SS-31 groups (*n* = 3–6/group; ***p < 0.001, one-way ANOVA with Tukey’s post-hoc test). Scale: 25 µm. **l**, **m** Representative SHG images and analysis of the percentage of centrally nucleated fibers from WT, *Kl*^*+/−*^ and *Kl*^*+/−*^+ SS-31 mice at 14 dpi (*n* = 3–4/group; *p < 0.05, **p < 0.01, one-way ANOVA with Tukey’s post-hoc test). Scale: 50 µm. **n** Quantification of myofiber cross-sectional area from WT, *Kl*^*+/−*^ and *Kl*^*+/−*^+ SS-31 groups (*n* = 3–4/group; *p < 0.05, **p < 0.01, one-way ANOVA with Tukey’s post-hoc test). **o** Hang impulse (calculated as hanging time × mouse weight) at 14 dpi, represented as a fold-change from 1 dpi score during the wire hang test (*n* = 3–6/group; *p < 0.05, ***p < 0.001, two-way ANOVA with Tukey’s post-hoc test). Each mouse was used as its own control in order to account for baseline variability (shown in Supplementary Figure 8). Data represented as mean ± SEM

What is the mechanism by which α-Klotho regulates mitochondrial function? The disrupted mitochondrial cristae structure within cells displaying decreased α-Klotho expression (Fig. 4a) suggests that α-Klotho is necessary for the maintenance of mitochondrial matrix integrity. Loss of matrix integrity disrupts mitochondrial respiration and induces the accumulation of reactive oxygen species (ROS). ROS accumulation, in turn, drives mtDNA damage and, ultimately, cellular senescence^50^. This led us to ask whether α-Klotho may preserve mitochondrial function through maintenance of mitochondrial ultrastructure. TEM analysis reveals a significantly greater number of vacuolated mitochondria with disrupted cristae in the MPCs of *Kl*^*+/−*^ mice (Fig. 5a, b).

Cardiolipin is an anionic phospholipid that is confined almost exclusively to the inner mitochondrial membrane where it is synthesized. It is required for the mitochondrial respiratory supercomplex assembly and activity, the disruption of which enhances the generation of mitochondrial ROS^25^. We found that cardiolipin content is depleted in MPCs isolated from *Kl*^*+/−*^ mice, as compared to wild-type counterparts (Fig. 5c, d). However, treatment with SS-31, a mitochondrially targeted peptide that mitigates cardiolipin peroxidation^25^, restored *Kl*^*+/−*^ MPC ROS generation, mtDNA damage and cellular bioenergetics to levels comparable to wild-type counterparts (Fig. 5a–i, Supplementary Figure 8). Administration of SS-31 to *Kl*^*+/−*^ mice also resulted in an increased number of MyoD^+^ cells at the site of injury, an enhanced regenerative index, and an increased myofiber cross-sectional area (Fig. 5j–n). Accordingly, *Kl*^*+/−*^ mice treated with SS-31 display an increased strength recovery after injury that parallels that of wild-type counterparts (Fig. 5o). Of note, treatment of wild-type mice with SS-31 did not alter strength recovery after injury when compared to saline-injected counterparts. Taken together, these findings suggest that the defect in MPC mitochondrial bioenergetics and the accumulation of mtDNA lesions accompanying a loss of α-Klotho are mediated by disruption of mitochondrial ultrastructure, ultimately resulting in dysfunctional muscle regeneration.

### Muscle regeneration is enhanced by α-Klotho supplementation

**Fig. 6:**
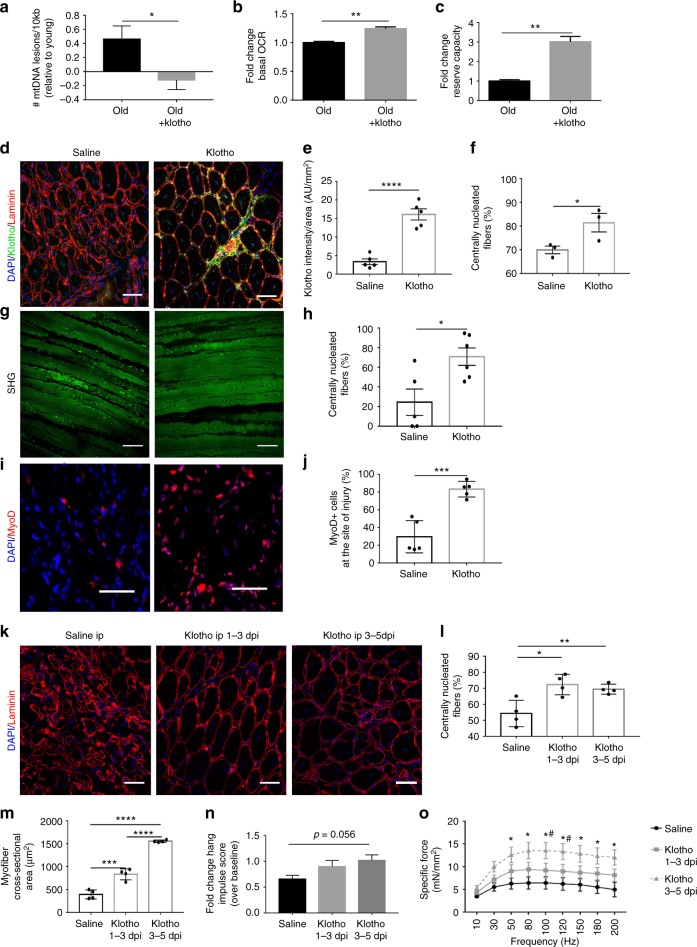
α-Klotho supplementation improves aged MPC bioenergetics and muscle regeneration. **a** RT-qPCR-based analysis of mtDNA damage in old and MPCs and in old MPCs that received supplementation with recombinant α-Klotho in the culture medium for 48 h (*n* = 3/group; *p < 0.05, one-tailed Student’s *t* test). **b**, **c** Seahorse analysis of basal OCR and reserve capacity of age and aged+ α-Klotho MPCs (*n* = 6–8/group; **p < 0.01). **d**, **e** Representative immunofluorescent images and quantification of α-Klotho expression in aged muscle 14 dpi after systemic supplementation of α-Klotho via an osmotic pump, as compared to saline-infused control muscles (*n* = 5/group; ****p < 0.0001). Scale: 50 µm. **f** Quantification of the percentage of centrally nucleated fibers as per histological analysis across saline and α-Klotho infused animals 14 dpi (*n* = 3/group; *p < 0.05). **g**, **h** Representative SHG imaging and quantification of the percentage of centrally nucleated fibers of saline versus α-Klotho infused animals at 14 dpi (*n* = 5–6/group; *p < 0.05). Scale: 35 µm. **i**, **j** Representative images and quantification of MyoD+ cells at the site of injury 14 dpi in animals receiving osmotic pump delivery of saline or α-Klotho (*n* = 5/group; ***p < 0.001). Scale: 25 µm. **k** Representative immunofluorescent images showing laminin and DAPI in animals receiving i.p. administration of saline, α-Klotho 1–3 dpi, and α-Klotho 3–5 dpi. Scale: 50 µm. **l** Quantification of percentage of centrally nucleated fibers in aged animals receiving i.p. administration of saline, α-Klotho 1–3 dpi, and α-Klotho 3–5 dpi (*n* = 4/group; *p < 0.05, **p < 0.01). **m** Quantification of fiber cross-sectional area across the three i.p. injection groups (*n* = 4/group; ***p < 0.001, ****p < 0.0001). **n** Fold change hang impulse score over baseline scores across the three i.p. injection groups (*n* = 6/group). **o** Force-frequency curves obtained from in situ contractile testing analysis of specific force (*n* = 6–8/group, *p < 0.05 when comparing α-Klotho 3–5 dpi with saline control, #*p* < 0.05 when comparing α-Klotho 3–5 dpi with α-Klotho 1–3 dpi group; two-way ANOVA with repeated measures). **a**−**j** One-tailed Student’s *t* test. **k**−**n** One-way ANOVA with Tukey’s post-hoc test. Data represented as mean ± SEM

Given the established hormonal role of α-Klotho, we next investigated whether supplementation of α-Klotho may restore mitochondrial function in aged MPCs. We found that when MPCs isolated from aged skeletal muscle were cultured in the presence of recombinant α-Klotho for 48 h, the aged mitochondrial phenotype was improved, as determined by decreased mtDNA damage, increased OCR, and increased reserve capacity (Fig. 6a–c).

These encouraging in vitro findings led us to next probe whether α-Klotho supplementation may enhance skeletal muscle regeneration in vivo. To do this, α-Klotho was administered to aged mice via osmotic pump. Osmotic pumps were implanted three days prior to injury and were maintained for 14 dpi. At the dose tested, we observed a significant increase in local α-Klotho within the injured muscle areas (Fig. 6d, e). Systemic administration of α-Klotho in aged muscle resulted in an increased number of regenerating fibers after injury, as determined both by histology and SHG imaging (Fig. 6d–h). These findings were consistent with an approximately 3.5-fold increase in the number of MyoD^+^ cells at the site of injury in animals that received supplementation with α-Klotho (Fig. 6i, j). However, osmotic pump delivery of α-Klotho yielded no significant increase in myofiber cross-sectional area or total muscle area when compared to saline counterparts.

Given that osmotic pump administration delivers α-Klotho continuously, we next tested the hypothesis that the timing of α-Klotho administration may be critical for functional tissue regeneration. To do this, we performed daily intraperitoneal injections of recombinant α-Klotho to aged mice either from 1–3 dpi or from 3–5 dpi (i.e. the timepoints at which we found *Klotho* to be highly expressed in young muscle (Fig. 1g)). Similar to our findings using osmotic pumps, α-Klotho delivery both from 1–3 dpi and 3–5 dpi resulted in an increased number of regenerating fibers when compared to vehicle controls (Fig. 6k, l). Both treatment groups also displayed an increased myofiber area, though the magnitude of the improvement was greater in mice receiving α-Klotho over 3–5 dpi (Fig. 6m). Importantly, only animals receiving α-Klotho over 3–5 dpi displayed an improved functional recovery (Fig. 6n, o, Supplementary Table 1).

Taken together, the data suggest that α-Klotho is required for an adequate regenerative response to an acute injury, and that supplementation with α-Klotho via the circulation promotes MuSC commitment and myofiber regeneration in aged mice when administered at the appropriate timepoint. These findings implicate declines in this longevity protein as a contributor to a defective muscle regenerative response with aging and raise the possibility of systemic administration of α-Klotho as a therapeutic approach to promote the healing of aged skeletal muscle after injury.

## Discussion

**Fig. 7:**
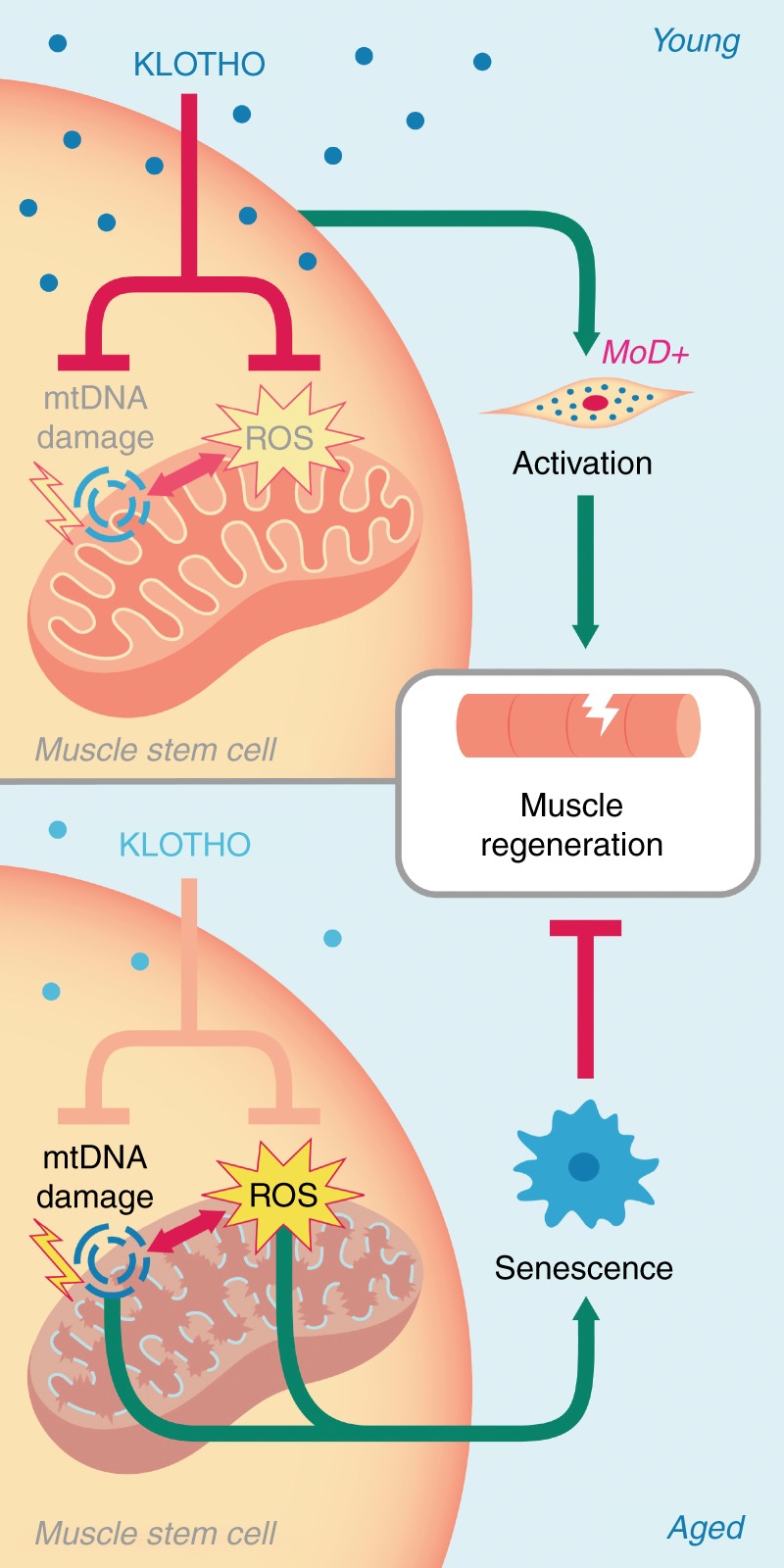
Hypothesis schematic. Youthful levels of the circulating hormone α-Klotho are critical for the maintenance of muscle stem cell (MuSC) mitochondrial ultrastructure, thereby limiting mtDNA damage and mitochondrial ROS production. This maintenance of healthy mitochondria within MuSCs is required for MuSC activation and contribution to functional skeletal muscle regeneration. However, age-related declines in α-Klotho causes disrupted mitochondrial ultrastructure, increased mtDNA damage, and ROS accumulation, resulting in cellular senescence and impaired skeletal muscle regeneration

Though aged muscle typically demonstrates a shift from functional myofiber repair following injury, to a quick-fix default towards fibrosis^5^, these age-related declines are reversible. Heterochronic parabiosis, in which circulatory systems of young and old animals are surgically joined, has clearly shown that systemic factors in young animals have a positive impact on the health of older animals^1,5,13,51^. Here, we tested the hypothesis that α-Klotho, a circulating hormone and paracrine factor associated with the attenuation of tissue aging is critical for effective MPC function and muscle regeneration after injury. We found that α-Klotho is highly upregulated at both the mRNA and protein level locally within the acutely injured muscles of young skeletal muscles. However, the response is markedly attenuated in aged muscles. At the cellular level, we uncovered a role for α-Klotho in the maintenance of mitochondrial ultrastructure, mtDNA damage, and MPC bioenergetics (Fig. 7). Though skeletal muscle α-Klotho expression under conditions of homeostasis is low^15^, the data indicate that expression of α-Klotho within injured muscle is required for functional regeneration. As an alternative, systemic supplementation with α-Klotho at physiologically relevant time points may promote muscle regeneration in aged mice.

Previous studies have demonstrated that decreased α-Klotho levels impair the wound healing response of the skin and small intestine through regulation of stem cell function^17^. In dystrophic muscle, α-Klotho levels are epigenetically repressed at the level of the *Klotho* promoter via DNA methylation and histone modification^26^. Our observation that *Klotho* is silenced in normal muscle but poised to lose the repressive marks following an injury presents a novel mechanism for regulating the contribution of local *Klotho* expression to regeneration after acute injury. It is intriguing that failure to derepress the *Klotho* promoter in the injury response contributes to the deficiency of aged muscle regeneration. This may have implications for mechanisms of degeneration or failed regeneration in other tissues where α-Klotho has been demonstrated to decline with age.

The impaired tissue healing previously observed in α-Klotho knockout mice is associated with decreased cellular proliferation^17,52^, decreased resistance to stress^52,53^, fibrosis formation^26^, and increased senescence^24^. Mitochondrial dysfunction is a hallmark of the aged tissue phenotype^54^, and, increasingly, mitochondria are emerging as a nexus that modulates cell-wide signaling pathways governing these cellular processes. Among these, Wnt/TGFβ, insulin growth factor (IGF)-1, and fibroblast growth factor (FGF) signaling, for example, have been shown to be responsive to signals emanating from the mitochondria, predominantly through ROS production^55–58^. It is noteworthy that these pathways have been implicated both in the antiaging effects of α-Klotho^17,30,59^ and in age-related declines in muscle regeneration^1,6,9,60,61^. A recent report demonstrated that α-Klotho inhibits Wnt signaling in aged MuSCs^27^. However, a direct role of α-Klotho on cellular mitochondrial function has remained poorly understood. While an impaired mitochondrial morphology with decreased α-Klotho levels has been previously demonstrated in α-Klotho knockout mice^62^, it is difficult to discern whether the mitochondrial defects are a primary result of α-Klotho deficiency, or if they represent a secondary consequence of cumulative tissue dysfunction in these progeroid mice.

It is plausible that the main actions of α-Klotho and FGF23 on phosphorous homeostasis that are lost in aging and in *Kl*^*+/−*^ mice promotes mitochondrial hyperpolarization and subsequent ROS production due to increased phosphate levels^63^. The studies presented here employed gain- and loss-of-function paradigms to demonstrate a direct role for α-Klotho in the maintenance of MPC mitochondrial morphology, genome integrity, and bioenergetics. Decreased levels of α-Klotho in MPCs and skeletal muscle were associated with vacuolated mitochondria possessing compromised cristae structure. This loss of architecture may lead to reorganization and oxidation of cardiolipin that, in turn, converts cytochrome *c* into an oxidant-generating peroxidase^25^. The reversal of the aged or *Kl*^*+/−*^ mitochondrial phenotype with SS-31 supports this mechanism, as SS-31 binding to cardiolipin protects the structure of mitochondrial cristae, prevents cardiolipin interactions with cytochrome *c*, and promotes oxidative phosphorylation^25^. SS-31 administration has also been shown to rejuvenate aged skeletal muscle mitochondrial energetics and performance^64^. It was recently shown that restoration of mitochondrial function by replenishing nicotinamide adenine dinucleotide (NAD+) levels protects aged MuSCs from senescence and enhances myogenicity^44^, further supporting the link between MuSC mitochondrial function and muscle regenerative capacity.

Consistent with the induction of an aged phenotype when α-Klotho expression is inhibited in young MPCs in vitro, depletion of α-Klotho in vivo impairs myofiber regeneration and drives fibrosis after an acute injury event. α-Klotho has been shown to regulate fibrosis formation under conditions of renal pathology, skin lesions, and muscular dystrophy^17,26,65^. The in vivo data suggest that, with acute injury, α-Klotho plays an important role in regulating physiological muscle regenerative cascade. It appears that only a portion of this role is attributed to α-Klotho within the muscle itself as local inhibition of α-Klotho results in an attenuated regenerative defect when compared to whole body knockdown. The fact that administration of α-Klotho enhanced MuSC lineage progression and myofiber regeneration in aged skeletal muscle suggests that supplementation via the circulation may be an effective means to compensate for the regenerative deficit observed in aged animals.

## Methods

### Animals

C57BL/6 young (4–6 months) and old (22–24 months) mice were received from the Jackson Laboratories or NIA rodent colony. *Kl*^*+/−*^ mice were obtained from MMRRC, UC Davis and were genotyped prior to inclusion in the studies. All procedures were approved by the Institutional Animal Care and Use Committee of the University of Pittsburgh. Animals were ear-tagged, randomly assigned to intervention group, and compared to age-matched littermate controls whenever possible. Mice were evaluated prior to inclusion in the study, and animals with obvious health problems were eliminated. Animal experiments were repeated across a minimum of two separate cohorts of the experimental groups. All primary endpoints were prospectively selected prior to analyses and investigators performing endpoint analysis were blinded to the experimental group whenever possible.

### Histological analysis of muscle regeneration

Wild-type male C57BL/6 young, *Kl*^*+/−*^ mice, or old mice received injuries to bilateral TA muscles via an intramuscular injection of cardiotoxin (10 µL of 1 mg/mL). Fourteen days following the injury, TAs were harvested for histological analysis of α-Klotho, fibrosis (Sirius red), degenerating myofibers (IgG), and myofiber regeneration (laminin). SHG imaging was performed on isolated TA muscles treated with an NTC or lentiviral knockdown of α-Klotho, as well as pump-administered animals in order to visualize collagen and myofibers within the muscle 14 dpi, as we previously described^66^.

### Primary muscle cell isolation

MPCs were isolated from young, *Kl*^*+/−*^, and aged mice, as previously described^66^. MuSCs were sorted using FACS for surface markers CD31−, CD45−, Sca1−, and VCAM+^29^. A modified protocol was used to isolate MuSCs and FAPs as CD31−, CD45−, α-7 integrin+ for MuSCs and CD31−, CD45− and α-7 integrin− for FAPs^67^. Gating strategy was based on sorting MuSCs and FAPs over a negative population of CD31 and CD45 (Supplementary Figure 9).

### Primary muscle cell imaging

Immunofluorescence staining (α-Klotho, Tom20 (mitochondrial marker), ki67, MyoD, Pax7 and HMGB1) and senescence-associated beta-galactosidase staining was performed in isolated cells. Transmission electron microscopy of fixed cells was performed, as previously described^66^. SIM was performed in young and old cells stained for α-Klotho and DAPI.

### ELISA

The levels of α-Klotho protein were measured by a colorimetric sandwich enzyme immunoassay (SEH757Mu, Cloud-Clone Corp), according to the manufacturer’s instructions. Each sample was measured in duplicate.

### Hanging-wire test

Strength endurance was tested using the hang-wire test, as in ref. ^68^. The Hang Impulse (HI) score was calculated as body weight (grams) **×** time hung (seconds). Male mice were used for all testing using C57Bl/6 mice. Wild-type and *Kl*^+/*−*^ were females for testing.

### Inhibition of and supplementation with α-Klotho

MPCs were treated with 25 nmol of silencing RNA (siRNA) to α-Klotho (GE Dharmacon, Product no.SO2462181G) for 48 h. As a control, young MPCs were treated with a nontargeting (scramble) siRNA. Aged MPCs were treated with 0.05 µg/mL exogenous α-Klotho (R&D Systems, Product no. aa 34–981), added to the culture media for 48 h.

### Epigenetic regulation of α-Klotho

At baseline, 3, 7, and 14 days after injury, TAs were snap frozen using liquid nitrogen for gene expression, methylation-specific PCR (MSPCR), and ChIP analysis, essentially as previously described^69^.

### MPC bioenergetics and mitochondrial DNA damage

OCR and extracellular acidification rate were measured in real time using a Seahorse XFe96 Extracellular Flux Analyzer (Billerica, MA) as previously described^45^. The basal OCR was measured by averaging the OCR values before treating the cells with oligomycin. Total reserve capacity was calculated by the differences of OCR between treatment with FCCP and 2DG and basal values. Mitochondrial DNA damage was quantified as previously described^47,49^.

### SS-31 administration

Isotonic saline or SS-31 (3 mg/kg dissolved in saline at 0.3 mg/mL) was administered daily via an i.p. injection to wild-type and *Kl*^*+/−*^ animals for the entire duration of injury^64^. For in vitro experiments, 100 nM of SS-31 was administered to MPCs isolated from *Kl*^*+/−*^ animals for 48 h. Dosing was based on studies demonstrating the effectiveness in a mouse model of chronic cardiomyopathy^70^ as well as in vitro dose ranging studies performed in C2C12s to evaluate inhibition of stress-induced mitochondrial membrane hyperpolarization and ROS generation.

### In vivo lentiviral knockdown of α-Klotho

In vivo α-Klotho knockdown was done using lentiviral vectors for a SMARTpool of 2.0×10^5^ TU/TA or 3.82×10^6^ TU/TA shRNA to α-Klotho per TA muscle. Given that there was no significant difference in the local α-Klotho expression between the two treatment groups, samples across the two treatment groups were pooled for analysis. Control animals received equal volumes of empty lentiviral vector. Knockdown was maintained for 3 weeks, after which time bilateral TAs were injured. Histology or SHG imaging was performed 14 days after injury.

### Supplementation of α-Klotho in vivo

Mini osmotic pumps containing either saline or α-Klotho (324 pg/mL in saline vehicle) were inserted subcutaneously into aged mice. After 2 days, bilateral TA muscles were injured by intramuscular CTX injection (as above). Osmotic pumps remained implanted until euthanasia 14 dpi. Isotonic saline or α-Klotho (10 µg/kg body weight) was administered to aged animals via daily intraperitoenal injections from  1–3 dpi or 3–5 dpi. The TAs were then harvested 14 dpi and preserved for histology or SHG analysis. Blood serum was also collected to evaluate circulating α-Klotho levels via ELISA. The activity of α-Klotho was confirmed as previously described^71^.

## Electronic supplementary material

Supplementary Information

Description of Additional Supplementary files

Supplementary Movie 1

Supplementary Movie 2

## Data Availability

The data supporting the results of this paper can be made available by the corresponding author upon reasonable request.

## References

[1] Brack AS (2007). Increased Wnt signaling during aging alters muscle stem cell fate and increases fibrosis. Science.

[2] Schultz E (1989). Satellite cell behavior during skeletal muscle growth and regeneration. Med. & Sci. Sports & Exerc..

[3] Schultz E, Gibson MC, Champion T (1978). Satellite cells are mitotically quiescent in mature mouse muscle: an EM and radioautographic study. J. Exp. Zool..

[4] Ryall JG, Schertzer JD, Lynch GS (2008). Cellular and molecular mechanisms underlying age-related skeletal muscle wasting and weakness. Biogerontology.

[5] Conboy IM, Conboy MJ, Smythe GM, Rando TA (2003). Notch-mediated restoration of regenerative potential to aged muscle. Science.

[6] Garcia-Prat L (2016). Autophagy maintains stemness by preventing senescence. Nature.

[7] Zerba E, Komorowski TE, Faulkner JA (1990). Free radical injury to skeletal muscles of young, adult, and old mice. Am. J. Physiol..

[8] Cosgrove BD (2014). Rejuvenation of the muscle stem cell population restores strength to injured aged muscles. Nat. Med..

[9] Bernet JD (2014). p38 MAPK signaling underlies a cell-autonomous loss of stem cell self-renewal in skeletal muscle of aged mice. Nat. Med..

[10] Price FD (2014). Inhibition of JAK-STAT signaling stimulates adult satellite cell function. Nat. Med..

[11] Tierney MT (2014). STAT3 signaling controls satellite cell expansion and skeletal muscle repair. Nat. Med..

[12] Sacco A, Puri PL (2015). Regulation of muscle satellite cell function in tissue homeostasis and aging. Cell Stem Cell.

[13] Conboy IM (2005). Rejuvenation of aged progenitor cells by exposure to a young systemic environment. Nature.

[14] Lukjanenko L (2016). Loss of fibronectin from the aged stem cell niche affects the regenerative capacity of skeletal muscle in mice. Nat. Med..

[15] Kuro-o M (1997). Mutation of the mouse klotho gene leads to a syndrome resembling ageing. Nature.

[16] Xiao NM, Zhang YM, Zheng Q, Gu J (2004). Klotho is a serum factor related to human aging. Chin. Med. J..

[17] Liu H (2007). Augmented Wnt signaling in a mammalian model of accelerated aging. Science.

[18] Semba RD (2011). Plasma klotho and cardiovascular disease in adults. J. Am. Geriatr. Soc..

[19] Shardell M (2016). Plasma Klotho and cognitive decline in older adults: findings from the InCHIANTI study. J. Gerontol. A Biol. Sci. Med. Sci..

[20] Semba RD (2016). Low plasma Klotho concentrations and decline of knee strength in older adults. J. Gerontol. A Biol. Sci. Med. Sci..

[21] Semba RD (2012). Relationship of low plasma klotho with poor grip strength in older community-dwelling adults: the InCHIANTI study. Eur. J. Appl. Physiol..

[22] Crasto CL (2012). Relationship of low-circulating “anti-aging” klotho hormone with disability in activities of daily living among older community-dwelling adults. Rejuvenation Res..

[23] Kuro-o M (2012). Klotho in health and disease. Curr. Opin. Nephrol. Hypertens..

[24] Kuro-o M (2008). Klotho as a regulator of oxidative stress and senescence. Biol. Chem..

[25] Szeto HH (2014). First-in-class cardiolipin-protective compound as a therapeutic agent to restore mitochondrial bioenergetics. Br. J. Pharmacol..

[26] Wehling-Henricks M (2016). Klotho gene silencing promotes pathology in the mdx mouse model of Duchenne muscular dystrophy. Hum. Mol. Genet..

[27] Ahrens HE, Huettemeister J, Schmidt M, Kaether C, von Maltzahn J (2018). Klotho expression is a prerequisite for proper muscle stem cell function and regeneration of skeletal muscle. Skelet. Muscle.

[28] van Velthoven CTJ, de Morree A, Egner IM, Brett JO, Rando TA (2017). Transcriptional profiling of quiescent muscle stem cells in vivo. Cell Rep..

[29] Cheung TH (2012). Maintenance of muscle stem-cell quiescence by microRNA-489. Nature.

[30] Kurosu H (2006). Regulation of fibroblast growth factor-23 signaling by klotho. J. Biol. Chem..

[31] Megeney LA, Kablar B, Garrett K, Anderson JE, Rudnicki MA (1996). MyoD is required for myogenic stem cell function in adult skeletal muscle. Genes Dev..

[32] Liu L, Cheung TH, Charville GW, Rando TA (2015). Isolation of skeletal muscle stem cells by fluorescence-activated cell sorting. Nat. Protoc..

[33] Joe AW (2010). Muscle injury activates resident fibro/adipogenic progenitors that facilitate myogenesis. Nat. Cell Biol..

[34] Wehling-Henricks M (2018). Macrophages escape Klotho gene silencing in the mdx mouse model of Duchenne muscular dystrophy and promote muscle growth and increase satellite cell numbers through a Klotho-mediated pathway. Hum. Mol. Genet..

[35] Sousa-Victor P (2014). Geriatric muscle stem cells switch reversible quiescence into senescence. Nature.

[36] Nassour J (2016). Defective DNA single-strand break repair is responsible for senescence and neoplastic escape of epithelial cells. Nat. Commun..

[37] Garreau-Balandier I (2014). A comprehensive approach to determining BER capacities and their change with aging in Drosophila melanogaster mitochondria by oligonucleotide microarray. FEBS Lett..

[38] Sauvaigo S (2004). An oligonucleotide microarray for the monitoring of repair enzyme activity toward different DNA base damage. Anal. Biochem..

[39] Chen KH (1998). Up-regulation of base excision repair correlates with enhanced protection against a DNA damaging agent in mouse cell lines. Nucleic Acids Res..

[40] Lopez-Otin C, Blasco MA, Partridge L, Serrano M, Kroemer G (2013). The hallmarks of aging. Cell.

[41] Kennedy BK (2014). Geroscience: linking aging to chronic disease. Cell.

[42] Fulco M (2008). Glucose restriction inhibits skeletal myoblast differentiation by activating SIRT1 through AMPK-mediated regulation of Nampt. Dev. Cell.

[43] Ryall JG (2015). The NAD(+)-dependent SIRT1 deacetylase translates a metabolic switch into regulatory epigenetics in skeletal muscle stem cells. Cell Stem Cell.

[44] Zhang H (2016). NAD(+) repletion improves mitochondrial and stem cell function and enhances life span in mice. Science.

[45] de Moura MB, Van Houten B (2014). Bioenergetic analysis of intact mammalian cells using the Seahorse XF24 Extracellular Flux analyzer and a luciferase ATP assay. Methods Mol. Biol..

[46] Furda AM, Marrangoni AM, Lokshin A, Van Houten B (2012). Oxidants and not alkylating agents induce rapid mtDNA loss and mitochondrial dysfunction. Dna Repair. (Amst.)..

[47] Sanders LH (2014). LRRK2 mutations cause mitochondrial DNA damage in iPSC-derived neural cells from Parkinson’s disease patients: reversal by gene correction. Neurobiol. Dis..

[48] Sanders LH (2014). Mitochondrial DNA damage: molecular marker of vulnerable nigral neurons in Parkinson’s disease. Neurobiol. Dis..

[49] Sanders LH, Howlett EH, McCoy J, Greenamyre JT (2014). Mitochondrial DNA damage as a peripheral biomarker for mitochondrial toxin exposure in rats. Toxicol. Sci..

[50] Yakes FM, Van Houten B (1997). Mitochondrial DNA damage is more extensive and persists longer than nuclear DNA damage in human cells following oxidative stress. Proc. Natl. Acad. Sci. USA.

[51] Sinha M (2014). Restoring systemic GDF11 levels reverses age-related dysfunction in mouse skeletal muscle. Science.

[52] Izbeki F (2010). Loss of Kitlow progenitors, reduced stem cell factor and high oxidative stress underlie gastric dysfunction in progeric mice. J. Physiol..

[53] Yamamoto M (2005). Regulation of oxidative stress by the anti-aging hormone klotho. J. Biol. Chem..

[54] Gonzalez-Freire M (2015). Reconsidering the role of mitochondria in aging. J. Gerontol. A Biol. Sci. Med. Sci..

[55] Wen JW, Hwang JT, Kelly GM (2012). Reactive oxygen species and Wnt signalling crosstalk patterns mouse extraembryonic endoderm. Cell. Signal..

[56] Papaconstantinou J (2009). Insulin/IGF-1 and ROS signaling pathway cross-talk in aging and longevity determination. Mol. Cell. Endocrinol..

[57] Lo YY, Cruz TF (1995). Involvement of reactive oxygen species in cytokine and growth factor induction of c-fos expression in chondrocytes. J. Biol. Chem..

[58] Rozo M, Li L, Fan CM (2016). Targeting beta1-integrin signaling enhances regeneration in aged and dystrophic muscle in mice. Nat. Med..

[59] Kurosu H (2005). Suppression of aging in mice by the hormone Klotho. Science (New York, NY).

[60] Yousef H (2015). Systemic attenuation of the TGF-beta pathway by a single drug simultaneously rejuvenates hippocampal neurogenesis and myogenesis in the same old mammal. Oncotarget.

[61] Chakkalakal JV, Jones KM, Basson MA, Brack AS (2012). The aged niche disrupts muscle stem cell quiescence. Nature.

[62] Murata M, Miwa Y, Sato I (2009). Expression of respiratory chain enzyme mRNA and the morphological properties of mitochondria in the masseter muscles of klotho mutant mice. Okajimas Folia Anat. Jpn..

[63] de Cavanagh EA, Inserra F, Ferder L (2015). Angiotensin II blockade: how its molecular targets may signal to mitochondria and slow aging. Coincidences with calorie restriction and mTOR inhibition. Am. J. Physiol. Heart Circ. Physiol..

[64] Siegel MP (2013). Mitochondrial-targeted peptide rapidly improves mitochondrial energetics and skeletal muscle performance in aged mice. Aging Cell.

[65] Zhou L (2013). Loss of Klotho contributes to kidney injury by derepression of Wnt/beta-catenin signaling. J. Am. Soc. Nephrol..

[66] Zhang Changqing, Ferrari Ricardo, Beezhold Kevin, Stearns-Reider Kristen, D'Amore Antonio, Haschak Martin, Stolz Donna, Robbins Paul D., Barchowsky Aaron, Ambrosio Fabrisia (2016). Arsenic Promotes NF-Κb-Mediated Fibroblast Dysfunction and Matrix Remodeling to Impair Muscle Stem Cell Function. STEM CELLS.

[67] Yi, L. & Rossi, F. Purification of progenitors from skeletal muscle. *J. Vis. Exp*. 10.3791/2476 (2011). 10.3791/2476 PMC3197294 21445045

[68] Aartsma-Rus, A. & van Putten, M. Assessing functional performance in the m*dx* mouse model. *J. Vis. Exp*. **85**, 10.3791/51303 (2014). 10.3791/51303 PMC4158772 24747372

[69] Lin AH, Shang Y, Mitzner W, Sham JS, Tang WY (2016). Aberrant DNA methylation of phosphodiesterase [corrected] 4D alters airway smooth muscle cell phenotypes. Am. J. Respir. Cell Mol. Biol..

[70] Dai DF (2011). Mitochondrial targeted antioxidant peptide ameliorates hypertensive cardiomyopathy. J. Am. Coll. Cardiol..

[71] Shalhoub V (2011). Fibroblast growth factor 23 (FGF23) and alpha-klotho stimulate osteoblastic MC3T3.E1 cell proliferation and inhibit mineralization. Calcif. Tissue Int..

