# Supplementary material for: Age-related declines in α-Klotho drive progenitor cell mitochondrial dysfunction and impaired muscle regeneration
Source: Nat Commun. 2018 Nov 19;9:4859. doi: 10.1038/s41467-018-07253-3 (PMC6242898; doi:10.1038/s41467-018-07253-3)
Supplement: Supplementary file 1 — Supplementary Information [file 41467_2018_7253_MOESM1_ESM.docx]

### Supplementary Information

**Age-related declines in** α**-Klotho drive progenitor cell mitochondrial dysfunction and impaired muscle regeneration**

## **Sahu A et. al.**

### List of contents:

### Supplementary Figures 1-9

### Supplementary Table 1

### Supplementary Methods

### References

**
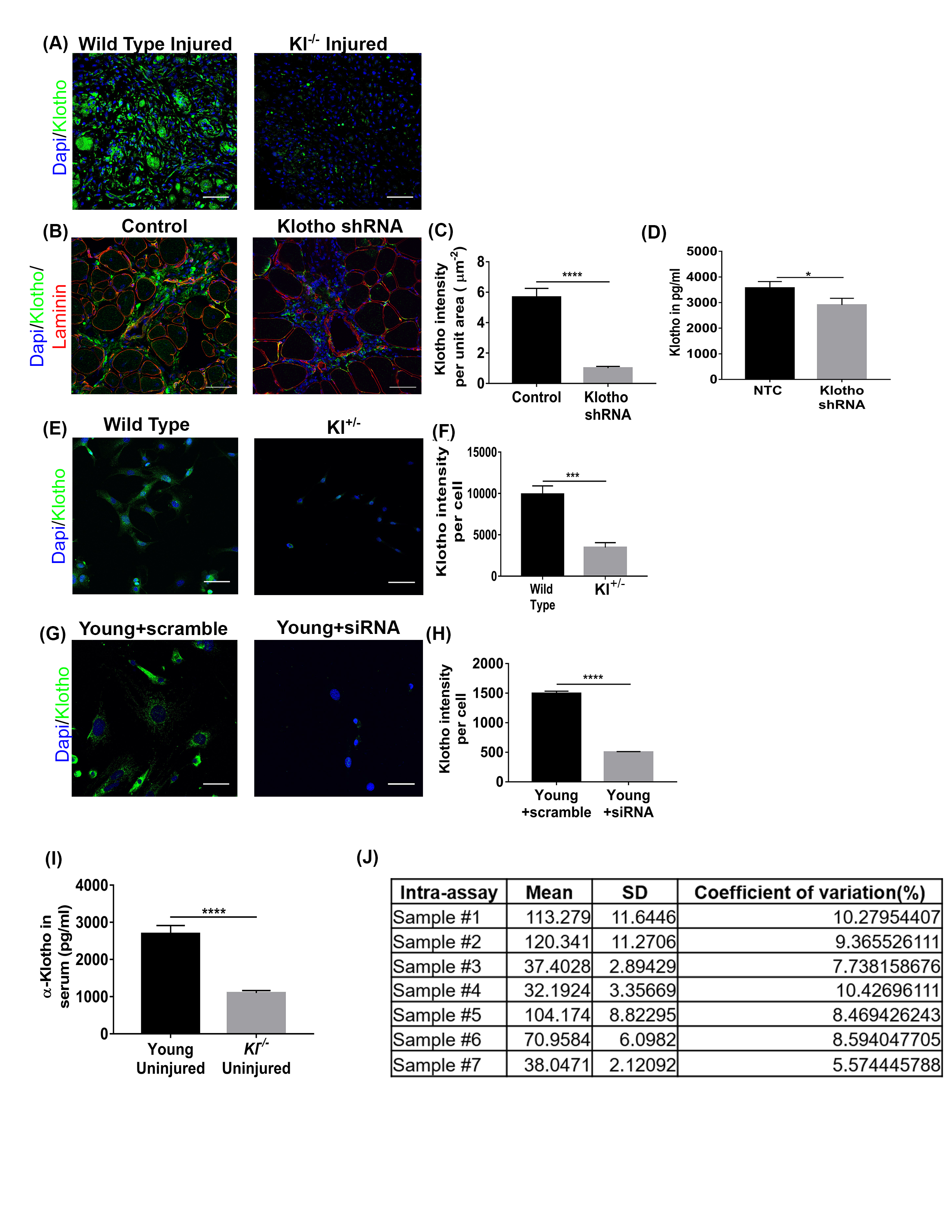
**

**Supplementary Figure 1. Antibody validation**. (A) To validate the antibody (MAB1819, R&D Systems) used for histology, muscle sections were co-stained for Klotho and DAPI in wild type and *Kl^-/-^* mice. Minimal α-Klotho was detected in the *Kl^-/-^* mice. Knockdown of α-Klotho by lentiviral shRNA revealed ~3-fold decrease in expression in the muscle (B, C) and a decline in circulating Klotho (D). (E, F) MPCs isolated from wild type and *Kl^+/-^* mice were co-stained for α-Klotho and DAPI. Immunofluorescence imaging revealed that MPCs from *Kl^+/-^* mice expressed ~50% less α-Klotho. (G, H) In the MPCs, α-Klotho knockdown using a siRNA, revealed ~3-fold decrease in α-Klotho expression. (I) The ELISA kit (Cloud-Clone Corp, SEH757Mu, Lot#L170622859) was validated by comparing serum levels of α-Klotho from young uninjured (n=8) and *Kl^-/-^* mice (n=6). Serum from *Kl^-/-^* mice revealed some non-specific binding of the target protein. (J) Intra-assay precision was determined by the coefficient of variation for 7 samples repeated in quintuplicate (*p<0.05, ***p<0.001, ****p<0.0001, student t-test). (A, B, E, G) Scale: 50µm. Data represented as mean + SEM.


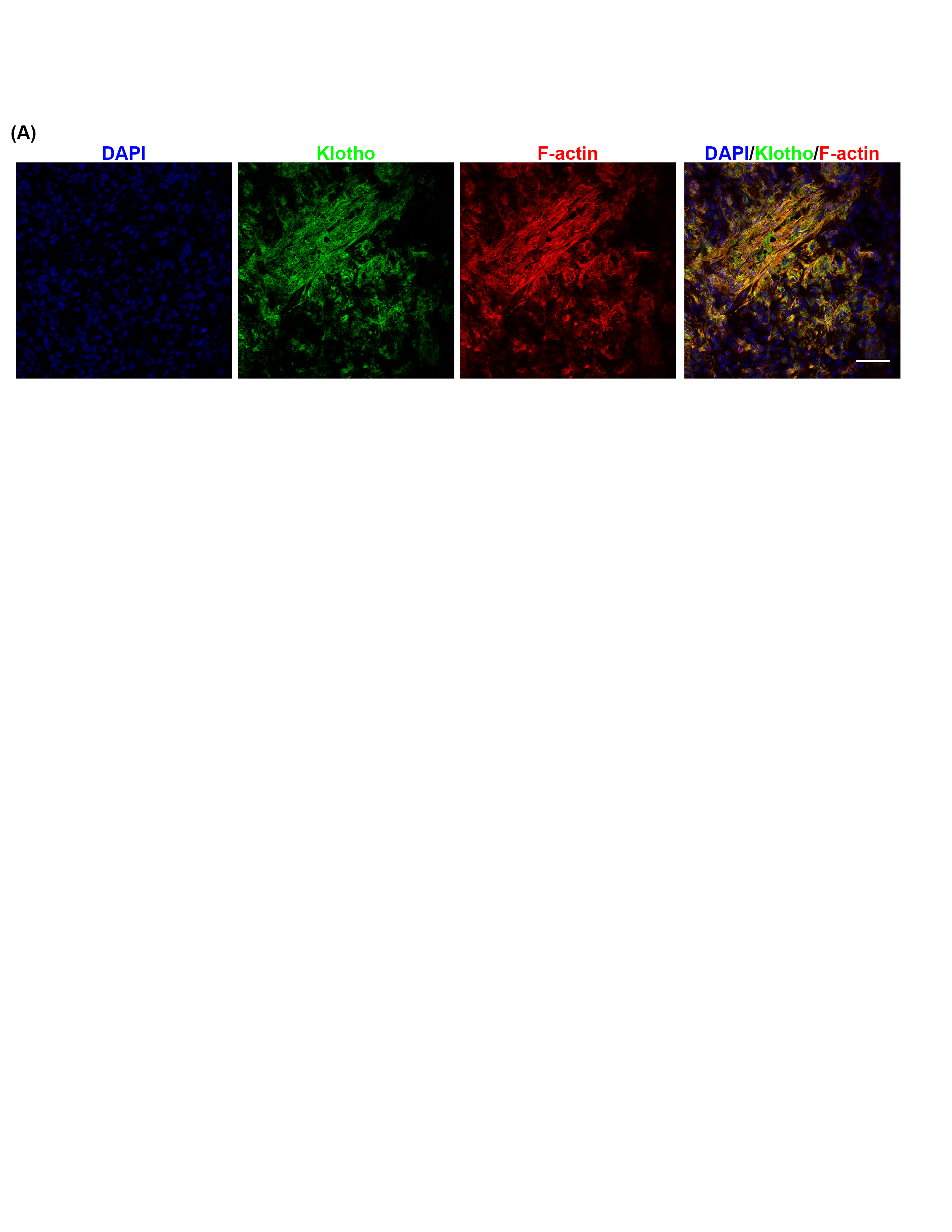


**Supplementary Figure 2. α-Klotho is also expressed in female muscle with a contusion injury**. (A) To confirm that α-Klotho’s response to injury is not unique to male mice or cardiotoxin injury, TA muscle sections from a female contusion model were co-stained for α-Klotho and F-actin and imaged using confocal microscopy (Scale: 50 µm).


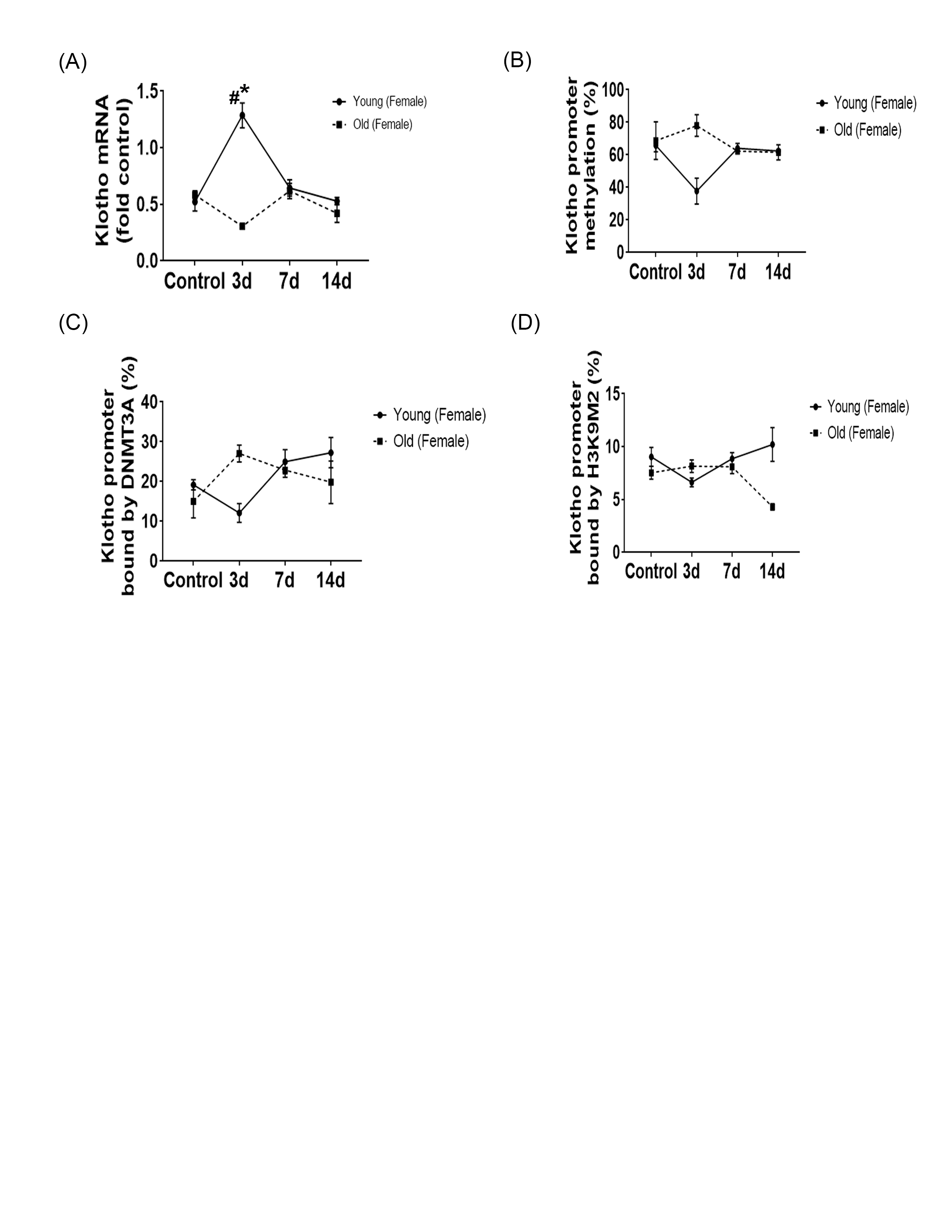


**Supplementary Figure 3. Aging results in a blunted *Klotho* response following injury in female mice**. (A) *Klotho* expression is increased in young females 3 days post injury (dpi), after which time levels return to the basal state. However, this response is blunted with aging. (B) Demethylation of the *Klotho* promoter occurs 3 dpi in young female mice, but the response is absent in aged female muscle. (C) There was a decrease in the DNMT3a binding in the young females which is returned to basal binding at 7 and 14 dpi. The reverse trend was observed in aged females. (D) H3K9M2 binding to *Klotho* promoter declined at 3dpi and then increased by 14 dpi. However, aged females displayed a decreased H3K9M2 binding to *Klotho* promoter at 14 dpi. (n=3/group/time point, *p<0.05 compared to young uninjured muscles; #p<0.05 indicates a significant difference between young and aged groups at the respective time-point, two-way ANOVA with tukey’s post hoc test). Data represented as mean + SEM.

**
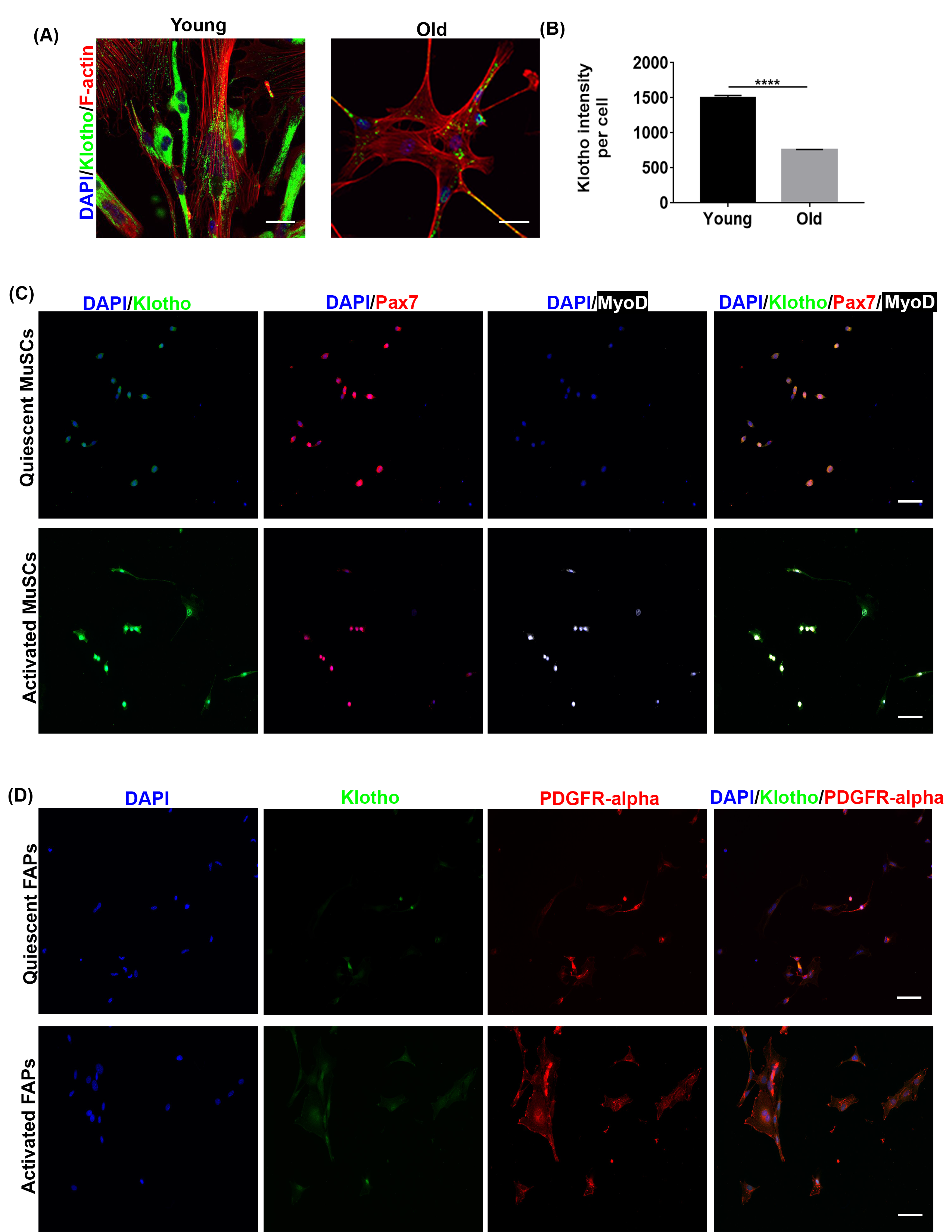
**

**Supplementary Figure 4. α-Klotho expression in MuSCs and FAPs**. (A, B) The intensity of α-Klotho was quantified in a purified population of flow-sorted muscle stem cells (MuSCs), which were subsequently cultured for 6 days. Aged MuSCs display significantly less α-Klotho when compared to young counterparts (Scale: 50 µm ; ****p<0.0001, Student’s t-test). (C) Flow sorted MuSCs express Pax7, MyoD and α-Klotho. (D) Confirmation of flow sorted FAPs expressing PDGFRα and α-Klotho. Scale: 50 µm. Data represented as mean + SEM.


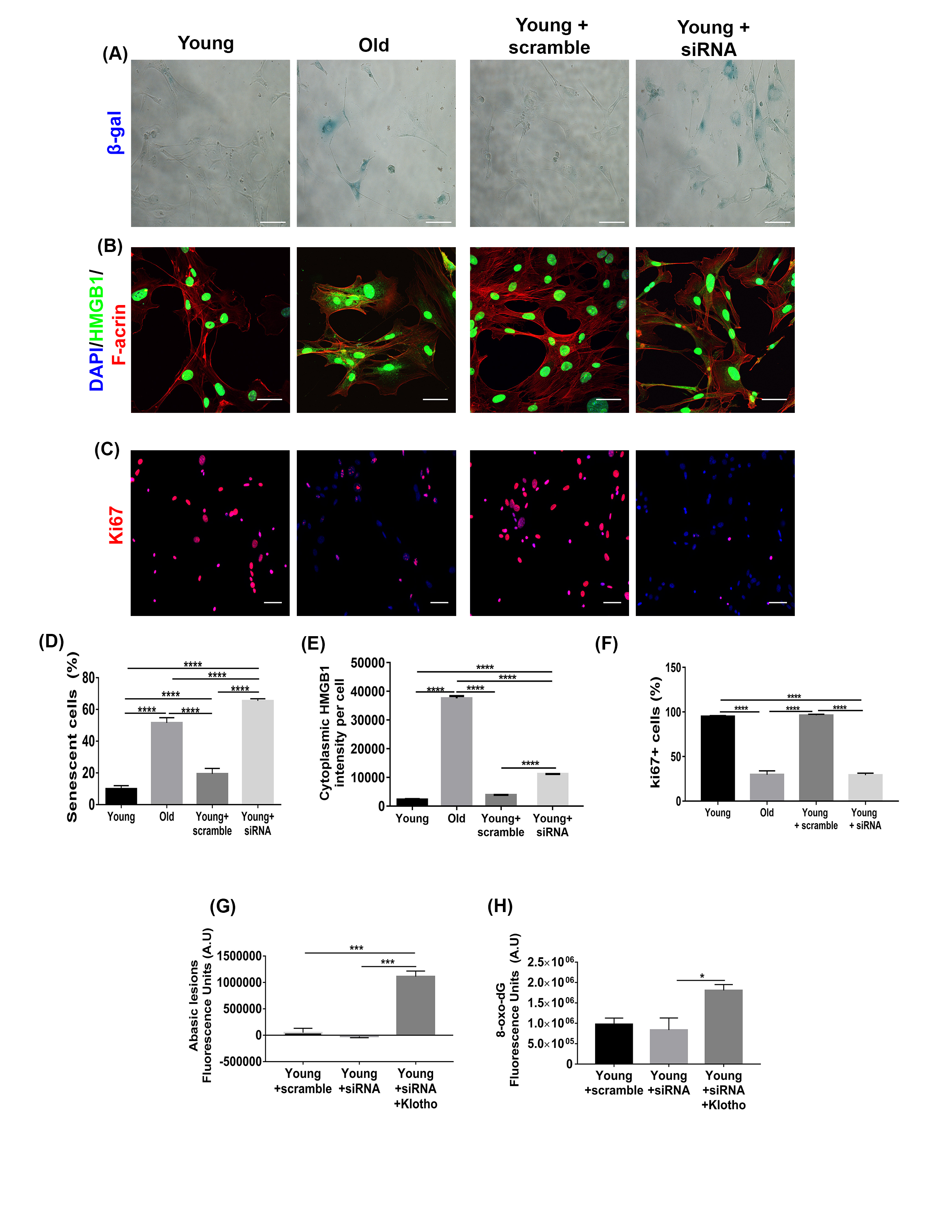


**Supplemental Figure 5. Decreased** α-**Klotho expression in MPCs is associated with increased cellular senescence.** Aged MPCs display increased senescence as evidenced by an increase in senescence-associated β-galactosidase expression (A, D; Scale: 100 µm) and increased cytoplasmic expression of HMGB1 (B, E; Scale: 50 µm). When young cells were treated with 25nmol of silencing RNA (siRNA) to α-Klotho, the average percentage of senescent cells was significantly higher when compared to young controls, as determined by SA-β gal and cytoplasmic HMGB1. There was no difference in the senescence profiles between old MPCs and young MPCs treated with siRNA to α-Klotho. (C, F) Inhibition of α-Klotho in young MPCs decreased cellular proliferation (Ki67 positivity) (Scale: 50 µm). Klotho supplementation in media while inhibiting Klotho using an siRNA stimulated repair of (G) abasic lesions (n=3) and (H) 8-oxo-dG (n=3). A minimum of 150 cells were analyzed per group for A-F. (****p<0.0001; ***p<0.001; *p< 0.05, one-way ANOVA with tukey’s post-hoc test). Data represented as mean + SEM.


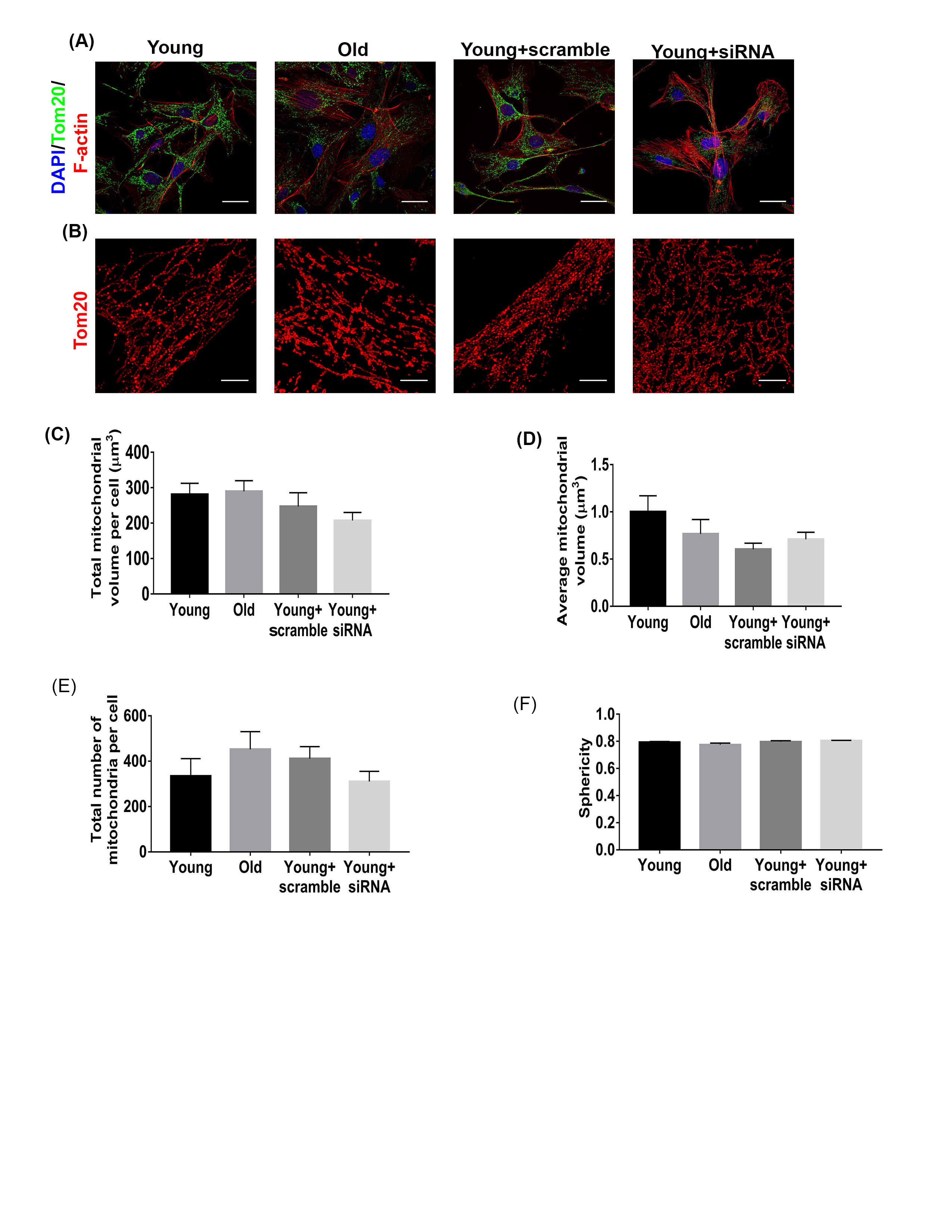


**Supplemental Figure 6. α-Klotho expression does not affect mitochondrial quantity or morphology**. (A, B) Confocal and STED microscopy of young, old, young+scramble and young+siRNA MPCs revealed that there is no difference in (C) total mitochondrial volume, (D) volume of each mitochondrion within a cell, (E) the number of mitochondria per cell in any of the groups, or the (F) mitochondrial sphericity (calculated as the ratio of the surface area of the given object to the surface area of a sphere with the same volume as the given object). At least 50 cells per group were analyzed. (p>0.05, one-way ANOVA with tukey’s post-hoc test). Data represented as mean + SEM.


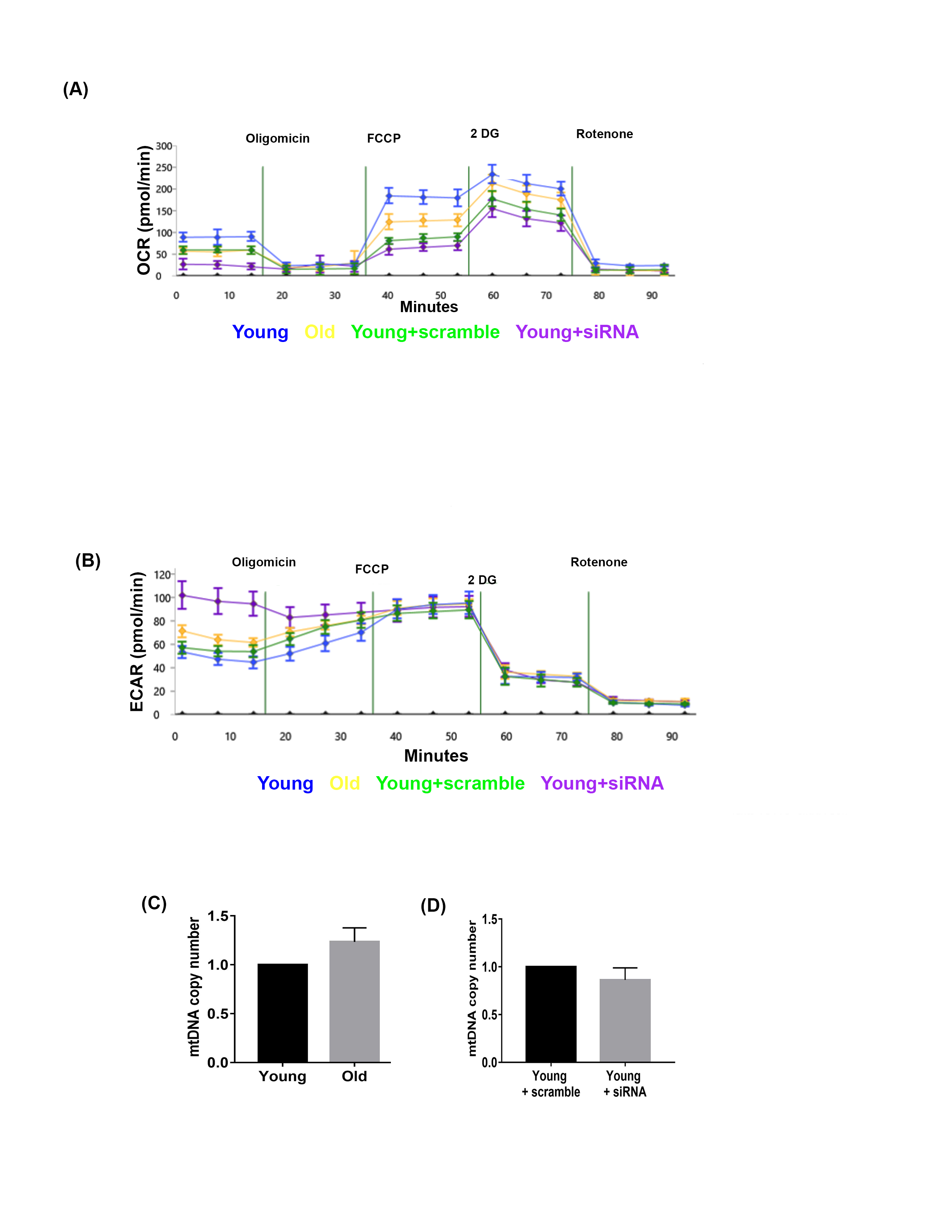


**Supplemental Figure 7: Expression of** α-**Klotho affects the bioenergetics profile of the cells but does not affect the mtDNA copy number**. (A, B) Representative bioenergetic profiles for oxygen consumption rate (OCR) and extracellular acidification rate (ECAR) of the MPCs. OCR and ECAR were quantified using a Seahorse XF_e_96 analyzer. These profiles are representative of eight separate biological repeat experiments performed in 4-6 replicates per run. (C, D) mtDNA copy number in MPCs is not altered with aging or when α-Klotho is knocked-down in young MPCs with an siRNA to α-Klotho. Data represented as mean + SEM.


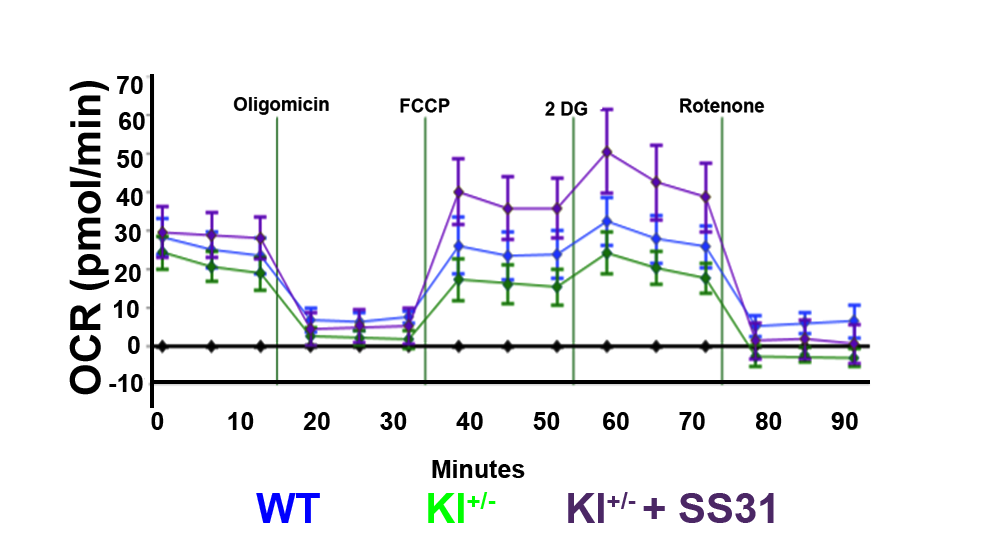

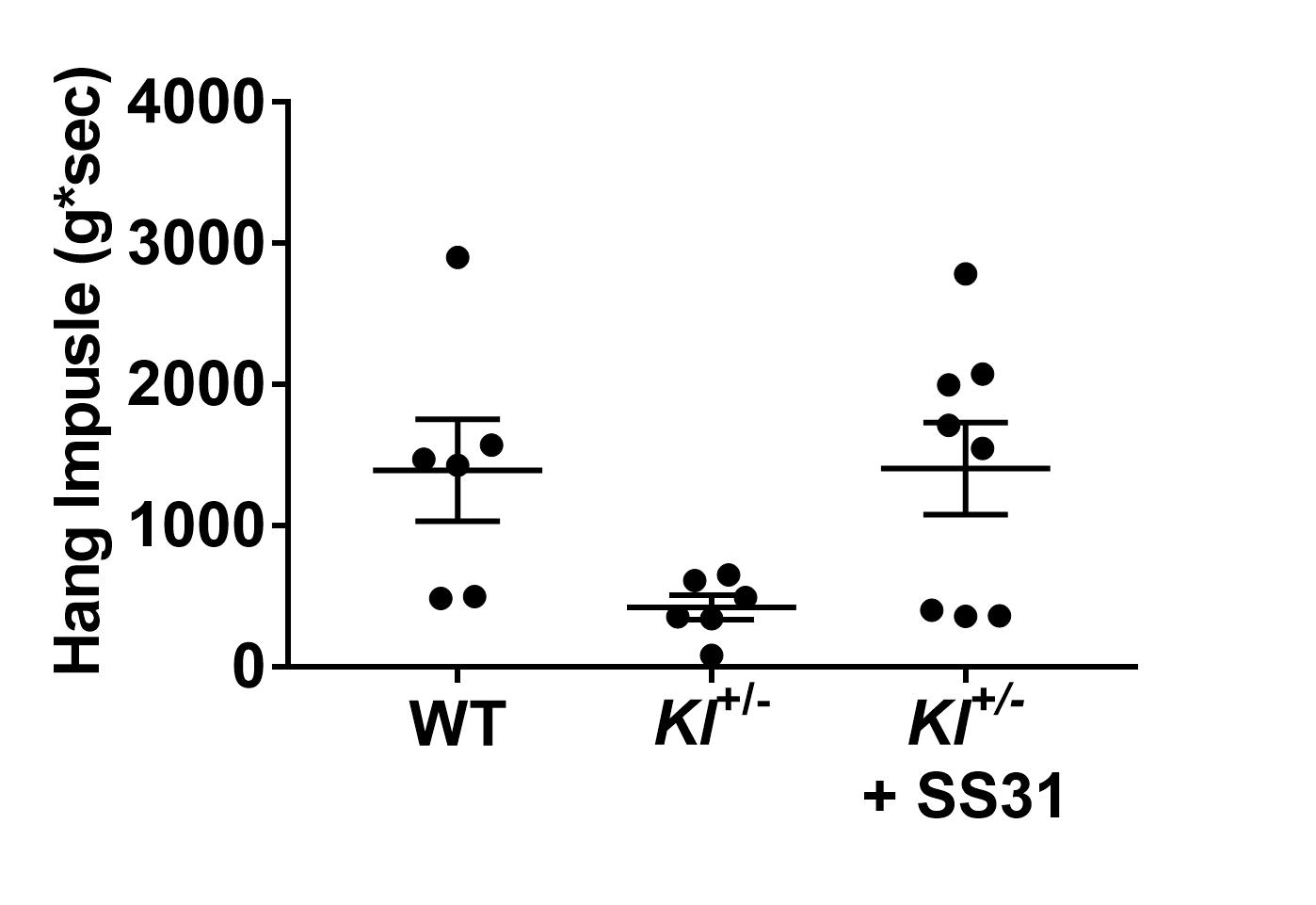


**(B)**

**(A)**

**Supplemental Figure 8. SS31 rescues the bioenergetics profile of *Kl^+/-^* MPCs to wild-type control levels, but does not significantly alter muscle strength in the absence of injury**. (A) Representative bioenergetic profiles for oxygen consumption rate (OCR) of the muscle myoblasts isolated from *Kl^+/-^* mice, as determined by Seahorse XF_e_96 analyzer. These profiles are representative of four separate biological repeat experiments performed in 4-6 replicates per run. (B) No significant differences (p>0.05, one-way ANOVA with tukey’s post-hoc test) were observed in the hang impulse (weight * number of seconds hanging on the wire) across the three experimental groups at baseline (i.e. prior to injury). Data represented as mean + SEM.

**(A)**

**(B)**

**(C)**


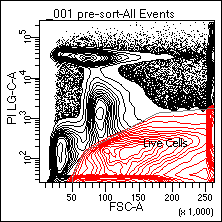

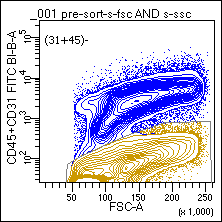

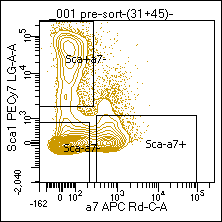


**Supplementary Figure 9. Representative gating strategy used to flow sort MuSCs and FAPs**. (A)  Sample was gated for live cells as well as a singlet discrimination gate based on pulse processing parameters.  (B) A negative population for CD31 and CD45 was gated on a forward scatter (FSC)/side-scatter (SSC) plot. (C) (CD31+CD45)- population was further gated to determine Sca1^-^+α7 integrin^+^ and Sca1^+^+ α7 integrin^-^ populations to yield MuSCs and FAPs, respectively.

| **Measurement** | **Saline** | **Klotho 1-3 dpi** | **Klotho 3-5 dpi** |
| --- | --- | --- | --- |
| Mice (n) | 8 | 6 | 6 |
| TA Weight (mg) | 46 ± 1.65 | 46 ± 1.42 | 46.5 ± 1.58 |
| Cross-sectional Area (mm^2^) | 2.80 ± 0.12 | 3.26 ± 0.06* | 3.30 ± 0.09* |
| Avg. Tetanic Torque (100 Hz, mN-m) | 0.94 ± 0.09 | 1.01 ± 0.14 | 1.38 ± 0.08* |
| Avg. Twitch Torque (mN-m) | 0.345 ± 0.012 | 0.422 ± 0.017* | 0.472 ± 0.018* |
| Avg. Specific Tetanic Force (100 Hz, mN/mm^2^) | 6.48 ± 1.37 | 9.21 ± 1.57 | 14.50 ± 1.78*# |
| Avg. Specific Twitch Force (mN/mm^2^) | 11.51 ± 1.06 | 14.08 ± 1.46 | 15.74 ± 1.62 |
| Time to Peak Twitch (s) | 0.026 ± 0.002 | 0.025 ± 0.001 | 0.024 ± 0.001 |
| 1/2 Relaxation Time (s) | 0.03 ± 0.004 | 0.036 ± 0.005 | 0.019 ± 0.001# |
| TA Length (mm) | 14.41 ± 0.15 | 13.31 ± 0.33* | 13.98 ± 0.26 |

**Supplementary Table 1. Skeletal muscle contractile characteristics.**

Values presented are mean + SEM. * indicates p<0.05 when compared to saline control, and # indicates p<0.05 when compared to Klotho 1-3 dpi.

**METHODS**

***Animal injury model and histological analysis of muscle regeneration***

All procedures were approved by the Institutional Animal Care and Use Committee of the University of Pittsburgh. Wild-type male C57BL/6 young (4-6 months, Jackson Laboratories), aged (22-24 months, NIA), *Kl* homozygotes (*Kl^+/+^*, B6; 129S5-Kltm1-Lex, 7-10 months, UC Davis) and *Klotho* heterozygotes (*Kl^+/-^*; B6; 129S5-Kltm1-Lex, 7-10 months, UC Davis) mice received injuries to bilateral tibialis anterior (TA) muscles via an intramuscular injection of cardiotoxin (CTX) (10 µL of 1 mg/mL cardiotoxin). Contusion injuries were performed as previously described (Ambrosio et al., 2009). For pain management, carprofen, an ingestible carprofen (Medigel), was provided for 3 days following injury. Fourteen days following injury, animals were euthanized, and the TAs were harvested for histological analysis. The TAs were fixed in 2% paraformaldehyde for one hour following which they were preserved in 30% sucrose overnight. Sucrose was changed 2-3 times. TAs were then frozen in liquid-nitrogen cooled 2-methylbutane and sectioned through their entirety into 10 µm sections. Muscle samples were not included in analyses if animals displayed evidence of external injuries and/or tumor growths upon euthanasia.

***Muscle harvest and serum isolation***

The tibialis anterior (TA) muscles were harvested using tweezers and scissors by removing the fascia around the muscle and cutting the proximal and distal tendons. Three, seven, or fourteen days after injury in young and aged animals, TAs were snap frozen using liquid nitrogen for PCR, methylation specific PCR, and ChIP analysis. Fourteen days after injury, the TAs were either preserved for histological analyses or for SHG imaging in ScaleView solution (Source: Olympus Miscroscope) for up to two weeks, after which time samples were transferred to a saline solution.

### For blood extraction, animals were placed in supine position while anesthetized by inhalation of isofluorane. The skin was cut from the abdomen to the neck and separated from the abdominal wall. The abdominal wall was then cut and the diaphragm was cut open for easy access to the apex of the heart. The chest cavity was then pulled open using a mosquito scissors. A 25 5/8 gauge, 1 mL needle was inserted into the apex of the heart, and the blood from the heart was drawn. To avoid hemolysis, the needle of the syringe was then removed, and the blood from the syringe was placed into a 1.5mL Eppendorf tube. Blood samples were kept at room temperature for 60 minutes, after which time samples were centrifuged for 20 minutes at 13,000 rpm. Using a 200 µL micro-pipette, the serum was pipetted out of the tube. Samples were preserved as aliquots of 50 µL for ELISA in the -20°C freezer. Any samples displaying hemolysis (as evidenced by pink/red coloration) were not included in the analysis.

***Real Time RT-PCR and methylation-specific PCR***

Frozen TA tissues were pulverized and tissue powders were homogenized in Trizol reagent (Invitrogen). RNA was isolated 1µg of total RNA was treated with DNaseI and reverse transcribed using iScript™ gDNA Clear cDNA Synthesis Kit (BIO-RAD, Hercules, CA). The mRNA levels of the *Klotho* gene were quantified by SYBR Green-based real-time PCR (qPCR) using SsoAdvanced™ Universal SYBR® Green Supermix (BIO-RAD, Hercules, CA). *Klotho* gene expression levels were normalized to the expression level of *Rpl44*, and the fold changes of *Klotho* relative to universal mouse reference RNA was calculated using 2^-ΔΔCt^ method. Each sample was measured in duplicates. Primers sequence were: Rpl44 (Forward: 5’-AGATGAGGCAGAGGTCCAA-3’and Reverse: 5’-GTTGTAAGAAAGGCGGTCA-3’); Dnmt1 (Forward: 5’- GTCGGACAGTGACACCCTTT -3’ and Reverse: 5’- TTTAGTGGGGCCCTTCGTG -3’) and Dnmt3a (Forward: 5’- GGGCCACACGGCAGAG-3’ and Reverse 5’-TGCCGTGGTCTTTGTAAGCA-3’).

CpG site-specific methylation of the *Kl* promoter was quantified using previously reported sequences for muscle DNA (21). Control DNA (fully methylated and fully unmethylated) were mixed in various concentration to serve as quantification standards when determining the percentage of DNA methylation following qPCR. The frozen TA muscles were pulverized under liquid nitogen, fixed with 1% formaldehyde for 15 minutes followed by quenching with 125mM glycine solution. Crosslinked protein/DNA complexs were pelleted and washed with PBS containing protease inhibitor cocktails. Chromatin isolation and immunoprecipitation was performed using ChIP-IT kit (Active Motif, Carlsbad, CA), following the manufacturer’s instructions. ChIP-validated antibodies against DNMT3A (39206, Active Motif, Carlsbad, CA), H3K9M2 (ab1220, Abcam, Cambridge, MA), or or nonspecific negative control mouse IgG (Active Motif, Carlsbad, CA) were used for immunoprecipitation of each sample. Non-immunoprecipitated chromatin was used as input. Following reverse cross-linking and elution of chromatin, DNA from each sample was purified with QIAquick PCR purification kit (Qiagen, Germantown, MD). Purified DNA sample concentrations were quantified by QubitiT DNA HS assay kit (Thermo Fisher Scientific, Waltham, MA). 10ng of purified DNA was used for PCR of the *Kl* promoter. The investigators performing the experiment were blinded to the hypotheses at the time of data analysis.

***DNA isolation, quantification and PCR-based assay for measuring mtDNA damage***

DNA isolation and quantification was performed using a high molecular weight genomic DNA purification kit according to the manufacturer's protocol (QIAGEN Genomic tip either 20/G or 100/G) and Quant-iT Picogreen dsDNA quantification. Following genomic DNA isolation, the purity and quality was assessed using a Nanodrop (ND-1000). A PCR-based assay was used to calculate mitochondrial DNA lesion frequency. Reaction mixtures used KAPA Long Range HotStart DNA Polymerase (KAPABiosystems) in a 96-well platform. Primers used for the mouse long amplicon are 5’-GCC AGC CTG ACC CAT AGC CAT AAT AT-3’ and 5’-GAG AGA TTT TAT GGG TGT AAT GCG G-3’, and the short amplicon are 5’-CCC AGC TAC TAC CAT CAT TCA AGT-3’ and 5’-GAT GGT TTG GGA GAT TGG TTG ATG T-3’. Each biological DNA sample was performed in triplicate.

***Lentiviral in-vivo knockdown of α-Klotho***

*In-vivo* knockdown of α-Klotho in the TAs of mice was done with a shRNA to α-Klotho using a lentiviral vector. Young wild-type mice received non-targeting control shRNA (Smartvector NON-targeting hCMV-TurboGFP Control particles, GE Dharmacon) to bilateral TAs. Young mice received a dose of 2 x 10^5^ TU/TA α-Klotho shRNA on their left TAs and a dose of 3.82 x 10^6^ TU/TA α-Klotho shRNA on their right TAs. α-Klotho shRNA used were a pool of three different clones (Smartvector Lentiviral Mouse KL mCMV-TurboGFP shRNA, Clone 11285258, Clone 11852825, Clone 17740025). Since there was no statistical significance between the α-Klotho knockdown using the two shRNA doses tested, the data obtained from the two doses of shRNA were combined for analysis.

***Primary muscle cell isolation***

Hindlimb muscles were isolated from young, aged, *Kl^+/+^* and *Kl^+/-^* mice using scissors and tweezers while ensuring that minimal fat, fascia or tendons were included in the tissue harvest. The muscles removed were washed in PBS to remove hair, debris and/or blot clots. After successive digestion of the muscle with 2 mg/mL Collagenase XI (Sigma, C-7657) for 1 hour, 2.4 U/mL Dispase (Life Technologies, Cat. No. 17105-041) for 45 minutes and 0.1% Trypsin (Life Technologies, Cat. No. 15400-054) for 30 minutes, the cell suspension was filtered through a 0.70 µm strainer and the homogenate was plated onto Collagen I (Sigma, C8919) coated flasks or 6-well plates. Muscle progenitor cells were expanded in high serum proliferation medium containing DMEM (Life Technologies, Cat. No. 11995-040), 20% FBS (Life Technologies, Cat. No. 10438026), 1% Pen/Strep (Life Technologies, Cat. No. 15140122) and 0.5% chick embryo extract (MP Biochemicals, Cat. No. 092850145). MPCs were cultured for a maximum of three passages for all experiments.

A purified population of muscle stem cells (MuSCs) were sorted by flow cytometry sorting for CD31^-^, CD45^-^, Sca1^-^ and VCAM^+^, as previously described (Liu et al, 2015). In addition, MuSCs were sorted according to the presence of CD31^-^, CD45^-^ , Sca1^-^ and α-7 integrin^+^, whereas FAPs were sorted on the basis of CD31^-^, CD45^-^, Sca1^+^ and α-7 integrin^-^ (Yi et al, 2011).

***Immunofluorescence***

Cells and muscle sections were fixed with warm 2% Paraformaldehyde for 15 minutes followed by a triple wash with PBS. They were permeabilized using 0.1% Triton-X for 15 minutes followed by a triple wash with PBS. They were blocked with 3% BSA and 0.1% Triton-X for 45 minutes. Primary antibodies such as rabbit anti-Tom20 (1:1000, SantaCruz Biotech, sc11415), rabbit anti-γH2AX (1:1000, ABCAM, ab11175), rabbit anti-Ki67 (1:1000, ABCAM, ab15580), rabbit anti-Laminin (1:1000, ABCAM, ab11575), rabbit anti-HMGB1 (1:1000, ABCAM, ab 18256), rabbit anti-MyoD (1:500), mouse anti-Pax7 (DSHB, 1:50) and rat anti-Klotho (1:400, R&D Systems, MAB1819, Lot# KGN0315101), were diluted in 3% BSA+5% Goat Serum+0.1% Triton-X overnight at 4°C. If antibodies produced in mouse were used (such as Pax7), 10% affinipure goat anti-mouse IgG Fab fragment was added during the blocking step.

After a triple wash with PBS, the samples were incubated with their respective secondary antibodies, goat-anti rat Alexa Fluor 488, goat anti-rabbit Alexa Fluor 546 and Phalloidin 647 (for F-actin) in 3% BSA+5% Goat Serum+0.1% Triton-X for 60 minutes. All secondary antibody dilutions were done at 1:500. Following a triple wash with PBS, the sampleswere stained with 0.02 mg/mL DAPI (Biolegend, Cat#422801) for 2 minutes and then washed with PBS again. The chamber sides and muscle sections were mounted with a glass coverslip using Gelvatol (Source: Center for Biologic Imaging (CBI), University of Pittsburgh) as a mounting media. These were dried in 4ºC for at least three hours before imaging.

***Confocal imaging***

Imaging was performed using a Nikon Confocal Microscope at 40X magnification at the Center for Biological Imaging (CBI) of the University of Pittsburgh. Muscle sections and chamber slides were imaged at a resolution of 1024x1024 and pixel depth of 1/8. Z-stacks were taken for rendering a 3-D picture of the sample with a slice depth of 0.5 µm.

***Second Harmonic Generation imaging***

Second Harmonic Generation (SHG) imaging was performed on TA muscles to visualize myofibers within the muscle. Muscle samples were first incubated at 4°C in ScaleView (CBI, Olympus) solution for at least one week to reduce opacity. Each muscle sample was rinsed with deionized water before being mounted on a custom glass plate with a v-shaped well to hold the sample in place. The well was then filled with deionized water and a cover slip was mounted. Correction for coverslip was applied and laser was set to 830 nm with 3.5-5% power. Z-stacks were taken with a thickness of roughly 180 μm from the 358 nm and 488 nm channels. Excitement wavelength for myofibers is 488nm. Investigators responsible for SHG imaging of the SS31 administration, lentiviral inhibition and the osmotic pump experiments, were blinded to the experimental groups for imaging as well as analysis.

***ELISA***

The levels of Klotho protein were measured by a colorimetric sandwich enzyme immunoassay (ELISA Kit SEH757Mu, Cloud-Clone Corp, Lot#L170622859), according to instructions of the kit. Briefly, for every experiment, standards and samples (Serum diluted 1:25 in PBS, 100μl/well) were added in duplicates to the 96 well microtiter plate that were pre-coated with a biotin-conjugated antibody specific for Klotho detection. The plates were incubated for one hour at 37°C. Subsequently, the samples and standard were removed. 100 µL of biotin-conjugated antibody was then added to each well and incubated for 1 hour at 37°C. The microplates were washed three times with washing buffer (2 minutes each wash), followed by the addition of 100 µL of avidin-conjugated HorseRadish-Peroxidase (HRP-avidin) to each well. The plate was incubated for 30 minutes at 37 °C after which the plate was washed five times with washing buffer. Next, 90ul of tetramethyl benzidine (TMB) substrate was added and the plate was incubated at 37 °C for 20 minutes. A sulfuric acid stop solution was then added to terminate color development reaction and the optical density (OD) of each well was measured at wavelength of 450nm. In order to determine antigen concentration of the unknown, an OD of the sample was compared to the OD standard curve generated using known antigen concentrations. The concentration range used for standard curve was 3.25 pg/mL to 200 pg/mL.

Samples displaying evidence of hemolysis or when insufficient quantities of serum were obtained were not included in data analysis, as we have found this to affect readout of Klotho levels. Media was collected after culturing MPCs for 3 days in 3 wells of a 12-well plate. For MuSCs and FAPs, media was collected after culturing them in 3 wells of chamber slides for 3 days. The data was then normalized to the number of cells per well. No samples were subjected to freeze-thaw, as we have observed that this may dramatically affect α-Klotho levels detected.

***In vitro inhibition of α-Klotho***

MPCs were plated onto collagen-coated flask and grown to 80% confluence. The cells were then treated with 25 nmol of silencing RNA (siRNA) to α-Klotho or a non-targeting scramble control (GE Dharmacon, Product no. SO2462181G) in antibiotic free proliferation media for 48 hours. Following treatment, cells were passaged and prepared for Transmission Electron Miscroscopy (TEM), STED microscopy, immunofluorescence (IF) staining, mtDNA damage analysis or Seahorse analysis. Cells were plated at a density of 10,000 cells per well of a collagen I coated chamber slide and grown for 24 hours for IF staining. They were plated at a density of 30,000 cells per well of a 96-well plate for seahorse experiments.

***Structured Illumination Microscopy***

Young and Old MPCs were plated on 35 mm glass bottom dishes (Matek, P35GCOL-0-10-C) at a density of 15,000 cells per dish for 48 hours. Cells were stained for α-Klotho and DAPI and preserved in PBS for imaging. The samples were illuminated with a patterned excitation light at 488 nm to detect Klotho and 405 nm to detect DAPI (nucleus). Samples were placed in a chamber on top of a 100X magnification oil lens. Type NF, Nikon immersion oil was used on the sample for imaging.

***Image Analysis***

ImageJ (1.49v, NIH, Bethesda) was used to quantify intensity of α-Klotho in cells and muscle sections along with the mitochondrial distribution within a cell. α-Klotho intensity was measured as integrated density of the pixels (i.e. intensity) in the α-Klotho channel. α-Klotho expression was normalized to either number of cells or to the total area of the image frame for muscle sections. For evaluation of α-Klotho intensity within the muscle, one image was obtained over 3-4 muscles sections at the maximal site of injury (identified as the muscle region containing the greatest number of centrally nucleated fibers and cellular infiltrate). Fibrosis was measured as the percentage of area covered by the sirius red stain under polarized light within the frame of injury. Cardiolipin and ROS were measured as a function of intensity of NAO and MitoSox per cell, respectively. Myofiber cross-sectional area was evaluated using ImageJ by manually tracing around the Laminin rings. Impaired mitochondria were evaluated using TEM images by quantifying the ratio of damaged mitochondria to intact mitochondria. Damaged mitochondria were identified to be swollen and vacuolated.

Imaris software was used to quantify mitochondrial volume, sphericity and volume of each mitochondrion in a cell. The investigator responsible for imaging mitochondria was blinded to the hypotheses at the time of data analysis. Regeneration index was also quantified using Imaris for the SHG images and ImageJ for histological images. Stereological analysis of regeneration was done by scanning through all slices of individual SHG z-stacks (over a depth of ~180 µm) and calculating the ratio of centrally nucleated fibers to total number of fibers. Investigators were blinded for imaging and data analysis.

***Transmission Electron Microscopy***

MPCs from each of the experimental groups were plated in a well of a plastic tissue culture 6-well plate and fixed in 2.5% Glutaraldehyde for 1 hour. In addition, TA muscles from wild type and *Kl^+/-^* mice treated with saline or SS-31 were fixed with 2.5% Glutaraldehyde for 24 hours. Following fixation, cell monolayers or muscle tissues were washed with PBS three times. Muscle samples were cut longitudinally into small pieces. The monolayers/tissues were then subjected to post-fixation aqueous solution of 1% osmium tetroxide, 1% Fe6CN3 for one hour. Samples were then washed in PBS three times then post-fixed in aqueous 1% osmium tetroxide, 1% Fe6CN3 for 1 hr. Following a triple wash with PBS, samples were dehydrated with a series of 30-100% ethanol and embedded in by inverting Polybed 812 (Polysciences, Warrington, PA) embedding resin-filled BEEM capsules on top of the monolayer. Blocks were cured twice, once overnight at 37°C, followed by curing at 65°C for two days. Post-curing, monolayers or tissues were peeled from the coverslip and cross-sectioned ultrathin (60 nm) on a Riechart Ultracut E microtome. The samples were then stained in uranyl acetate for 10 min and 1% lead citrate for 7 minutes. The investigators performing imaging were blinded to the hypotheses at the time of data analysis.

***Analysis of MPC bioenergetics***

MPCs from each of the experimental groups were plated on a 96-well plate at a density of 30,000 cells per well. CellTak was used as a cell adhesive. The cells were then cultured in un-buffered DMEM for 1 hour at 37˚C without CO_2_. The cells were then stressed by the successive injections of 1 µM Oligomycin, 300 nM FCCP (Carbonyl cyanide-4-(trifluoromethoxy)phenylhydrazone), 100 mM 2-DG (2-Deoxy-D-glucose) and 1 µM Rotenone. All reagents were prepared in unbuffered DMEM. The basal oxygen consumption rates (OCR, pmol/min) were plotted for each cell type by averaging the baseline levels before the first treatment with Oligomycin. The reserve capacity (pmol/min) was plotted by calculating the point-to-point difference between the maximum OCR levels (between treatment with FCCP and 2-DG) and basal OCR levels. The investigator performing the seahorse experiments was blinded to the hypotheses at the time of data analysis.

***MitoSox Live cell imaging***

MPCs isolated from wild type and *Kl^+/-^* mice were grown in collagen-coated flasks. Cells from *Kl^+/-^* mice were treated with SS-31. The three groups of cells (wild-type, *Kl^+/-^* and *Kl^+/-^* + SS-31) were then plated on individual 35 mm glass bottomed dishes (MatTek, P35GCOL-0-10-C) at a density of 30,000 cells per well. Cells were subsequently incubated for 24 hours, after which time cells were incubated with 5 µM MitoSox^TM^ reagent (Invitrogen, M36008) for 10 minutes in HBSS with Calcium and Magnesium. After the incubation, the cells were imaged live with an excitation/emission maxima of 510/580 nm on the Nikon Eclipse Ti Live Cell microscope (Nikon, CBI). The superoxide content was measured as a function of intensity per cell using Nikon’s NIS Elements software. For quantification purpose, at an average 200 cells per group were analyzed.

***Nonyl Acridine Orange (NAO) staining***

MPCs isolated from wild type and *Kl^+/-^* mice were grown in collagen-coated flasks. Cells from *Kl^+/-^* mice were treated with SS-31 or a vehicle (saline) control. Next, the three groups of cells (wild-type, *Kl^+/-^* and *Kl^+/-^* + SS-31) were plated on chamber slides at a density of 10,000 cells per well and were incubated for 24 hours. Cells were then fixed with 2% paraformaldehyde for 10 minutes, followed by 0.1% Triton-X for 10 minutes. The cells were then stained with 5 µM NAO for 15 minutes, which was diluted in HBSS (-Ca^2+^, -Mg^2+^) followed with triple PBS wash. The chamber slides were stained with DAPI for 2 minutes and then washed with PBS again. The chamber sides were mounted with a glass coverslip using Gelvatol. The cardiolipin content within a cell was measured as a function of intensity of NAO stain per cell.

***SS-31 administration***

A modified protocol was used to administer SS31 *in vivo* (Siegel et al, 2013). Isotonic saline or 3 mg/kg SS31 (SS-31 (NH2-(D)-Arg-(L)-2,6-Dimethyl Tyrosine-(L)-Lys-(L)-Phe–CONH_2_) was synthesized in the University of Pittsburgh Peptide Synthesis Core) dissolved in saline and was administered daily via i.p. injections to wild-type and *Kl^+/-^* animals for 17 days. The animals were injured with cardiotoxin 3 days after the i.p. injections started. They were subjected to a hanging-wire test and an *in situ* contractile testing 14 dpi for functional testing. Primary muscle progenitors were isolated from the euthanized mice using previously described method. The cells were then used for differentiation assay.

*In vitro,* SS-31 was administered to MPCs isolated from *Kl^+/-^* mice at a concentration of 100 nM for 48 hours. Three groups of cells: *Kl^+/+^, Kl^+/-^* and *Kl^+/-^* + SS31 were then analyzed for ROS production (MitoSox), bioenergetics profile, cardiolipin content and mtDNA damage.

***RNAseq gene expression analysis***

Paired end sequences were downloaded from Gene Expression Omnibus (GEO), a public database repository (GSE97399; van Velthoven et. al., 2017). NGS tool of CLC Genomics work-bench software (Qiagen) was used to perform quality control (QC) analysis for assessing quality indicators of the sequences on the basis of FastQC-project. Quality was assured by evaluating sequence-read lengths and base coverages; nucleotide contributions and base ambiguities; and quality scores that were emitted by the base caller and over-represented sequences. All samples that were analyzed, passed QC parameters and were then mapped to the annotated murine reference genome (Mus_musculys-enembl_v90). RNA-Seq analysis was performed using the transcriptomics analysis tool. Gene expressions were normalized by Transcripts Per kilobase Million (TPM). This method normalizes for the gene length first, following which it is normalized for sequencing depth second such that the sum of all TPMs in ever sample is the same. This allows for comparing proportion of reads that are mapped to a particular gene in each sample. Finally, a 1-Pearson correlation distance and complete linkage rule (Clus Vis package version 1.1.0) were used to provide unsupervised clustering for generating a heat-map.

RNAseq data that support the findings of this study have been queried from the publicly available NCBI database Sequence Read Archive (SRA) with the accession codes PRJNA381694; GEO: GSE97399 (https://www.ncbi.nlm.nih.gov/gds/?term=GSE97399)

***Supplementation with exogenous α-Klotho in vitro***

Aged MPCs were treated with 0.05 µg/mL of recombinant Klotho (R&D Systems, Product# aa 35-982) after which time they were grown to 60% confluence. The treatment was performed in antibiotic free proliferation media for 48 hours. The cells were then passaged and prepared for TEM, immunofluorescence staining, mtDNA damage analysis or Seahorse experiments. Cells were plated at a density of 10,000 cells per well and grown for 24 hours in a collagen I coated chamber slide for IF staining. They were plated at a density of 30,000 cells per well of a 96-well plate for seahorse experiments.

***Systemic supplementation with α-Klotho via osmotic pumps***

Osmotic pumps (Alzet, mini-osmotic pump, model#2004) were used to chronically deliver saline or 324 ng/mL α-Klotho (R&D Systems, Product# aa 35-982) to aged mice. Pumps were inserted subcutaneously at the back of the neck of mice, and the skin was stapled using 7 mm stainless steel wound clips (Reflex Skin Closure System, Reflex 7, Part no. 203-1000, Stoelting). For pain management, the mice were fed Carprofen as gel food (Medigel^R^, Clear H_2_O). Two days after implantation, bilateral TAs were injured using CTX. Tissues were harvested 14 days after injury for SHG imaging and histological analyses. Blood was collected to perform ELISA to test for α-Klotho levels in serum.

***Intraperitoneal administration of α-Klotho***

Isotonic saline or α-Klotho (10 µg/kg body weight; R&D Systems, Product# aa 35-982) was administered to aged animals via i.p. injections on days 1-3 post-injury or 3-5 days post-injury. We confirmed the activity of the recombinant α-Klotho prior to administration, as per a previously reported protocol (Shalhoub et al., 2011)

***Hanging-Wire Test***

A modified four-limb wire hanging test was used to measure mouse muscular endurance non-invasively and thus characterize performance of injured mice before and after any experimental intervention (Aartsma-Rus and van Putten, 2014). Mice were suspended upside down from a steel mesh grid (1 cm x 1 cm squares) above a custom built chamber (~30 cm high) with appropriate padding to prevent harm for falling down. Four-limb strength was evaluated using a Hang Impulse (HI) score (*bodyweight in grams* **x** *time hung in sec*).   Individual mice were subjected to five trials in one session with a gap of 5 minutes between trials, excluding the performance of other mice. Sessions were conducted at the same time of day and in the same order across trials. Evaluation of performance considers the average HI of three trials excluding the best trial and the worst trial for every mouse.

***In situ contractile testing***

*In situ* contractile testing of the TA muscles was performed as per a previously described protocol (Zhang et al., 2015). Briefly, the peroneal nerve was isolated by making an incision lateral to the knee. After cutting the Achilles tendon, the foot was placed on the force transducer and stabilized with a cloth tape over the foot. The needle electrodes were then placed on the peroneal nerve beneath the skin. Muscle peak tetanic force was evaluated with the ankle placed at 20° plantarflexion. The stimulator (Aurora Scientific, Model 701C) was used to elicit tetanic contractions at 10, 30, 50, 80, 100, 120, 150, 180 and 200 Hz, with a 2-minute rest between each contraction. From these data, the force-frequency curve was obtained. Results were collected in torque (mN-m). Force output (mN) was calculated by dividing the torque by the foot-plate length (0.03m). The specific tetanic force output at each frequency was then measured by normalizing each force output by the mean cross-sectional area (CSA, mm^2^). The mean CSA was calculated as the [muscle weight (mg)]/[muscle length (mm)×muscle density (1.06 mg/mm^3^)].

***Antibody validation***

We validated the α-Klotho antibody (R&D systems, MAB1819, Lot# KGN0315101) used for immunofluorescence by staining the injured (7 days) muscle section from a wild type and a *Kl^-/-^* mouse. We observed minimal background staining in the *Kl^-/^*^-^ muscle section as compared to the wild-type counterparts. Antibody was also validated by knocking down α-Klotho using an siRNA or shRNA to α-Klotho in MPCs and young TA muscle, respectively.

To validate the ELISA kit, serum from *Kl^-/-^* mice were compared to young uninjured controls. Intra-assay precision was quantified by calculating the coefficient of variation (ratio of standard deviation to mean) within the experiment.

***Statistical analysis***

All data are shown as mean ± SEM. Data were tested for normality by Shapiro-Wilk’s test. Bartlett’s-test was used to test for differences in variances. When the data were normally distributed, an independent one-way or two-way repeated measures ANOVA was used to compare differences among different post-injury groups within the same age group or between experimental groups at different time points, followed by Tukey’s post-hoc test, as appropriate. If data were not normally distributed, a Kruskal-Wallis test was performed for comparing between groups. A one-tailed unpaired student t-test was used for comparison between two groups, if data were normally distributed and the standard deviations (SD) were similar. For different SD, a welch’s correction was applied to the t test. If data were not normally distributed while comparing between two groups, the statistical differences were obtained by performing a Mann-Whitney U test. Differences between groups were considered significant at p < 0.05. Initial sample sizes were based on preliminary data, with an estimated effect size of 0.6, which yielded a sample size of 6 mice/group after adjusting for death and unforeseen circumstances (conventionally estimated at 20%).

***References***

AARTSMA-RUS, A. & VAN PUTTEN, M. 2014. Assessing functional performance in the mdx mouse model. *J Vis Exp*.

AMBROSIO, F., FERRARI, R. J., DISTEFANO, G., PLASSMEYER, J., CARVELL, G. E., DEASY, B. M., BONINGER, M. L., FITZGERALD, G. K. & HUARD, J. 2009. The Synergistic Effect of Treadmill Running on Stem Cell Transplantation To Heal Injured Skeletal Muscle. *Tissue Eng Part A*.

SAARMAN, N. P., KOBER, K. M., SIMISON, W. B. & POGSON, G. H. 2017. Sequence-Based Analysis of Thermal Adaptation and Protein Energy Landscapes in an Invasive Blue Mussel (Mytilus galloprovincialis). *Genome Biol Evol,* 9**,** 2739-2751.

SHALHOUB, V., WARD, S. C., SUN, B., STEVENS, J., RENSHAW, L., HAWKINS, N. & RICHARDS, W. G. 2011. Fibroblast growth factor 23 (FGF23) and alpha-klotho stimulate osteoblastic MC3T3.E1 cell proliferation and inhibit mineralization. *Calcif Tissue Int,* 89**,** 140-50.

ZHANG, C., FERRARI, R., BEEZHOLD, K., STEARNS-REIDER, K., D'AMORE, A., HASCHAK, M., STOLZ, D., ROBBINS, P. D., BARCHOWSKY, A. & AMBROSIO, F. 2015. Arsenic Promotes NF-kappaB-Mediated Fibroblast Dysfunction and Matrix Remodeling to Impair Muscle Stem Cell Function. *Stem Cells*.
