# Supplementary material for: Age-related declines in α-Klotho drive progenitor cell mitochondrial dysfunction and impaired muscle regeneration
Source: Nat Commun. 2018 Nov 19;9:4859. doi: 10.1038/s41467-018-07253-3 (PMC6242898; doi:10.1038/s41467-018-07253-3)
Supplement: Supplementary file 2 — Description of Additional Supplementary files [file 41467_2018_7253_MOESM2_ESM.pdf]

Legends to the Supplementary Movies are described below:

Supplementary Movie 1: 3-D visualization of an injured tibialis anterior muscle treated with a non-targeting control vector
Three-dimensional rendering of injured skeletal muscle treated with non-targeting control vector using Second Harmonic Generation imaging. The auto-fluorescence of muscle fibers is pseudo-colored in green and collagen is pseudo-colored in red.

Supplementary Movie 2: 3-D visualization of an injured tibialis anterior muscle treated with α-Klotho shRNA
Three-dimensional rendering of injured skeletal muscle treated with shRNA to α-Klotho using Second Harmonic Generation imaging. The auto-fluorescence of muscle fibers is pseudo-colored in green and collagen is pseudo-colored in red.
